# Diagnostic performance of AI-assisted endoscopy diagnosis of digestive system tumors: an umbrella review

**DOI:** 10.3389/fonc.2025.1519144

**Published:** 2025-04-03

**Authors:** Changwei Huang, Yue Song, Jize Dong, Fan Yang, Jintao Guo, Siyu Sun

**Affiliations:** ^1^ Department of Gastroenterology, Shengjing Hospital of China Medical University, Shenyang, Liaoning, China; ^2^ Engineering Research Center of Ministry of Education for Minimally Invasive Gastrointestinal Endoscopic Techniques, Shenyang, Liaoning, China

**Keywords:** artificial intelligence, endoscopy, endoscopic ultrasound, precancerous lesion, digestive system tumors

## Abstract

The diagnostic performance of artificial intelligence (AI)-assisted endoscopy for digestive tumors remains controversial. The objective of this umbrella review was to summarize the comprehensive evidence for the AI-assisted endoscopic diagnosis of digestive system tumors. We grouped the evidence according to the location of each digestive system tumor and performed separate subgroup analyses on the basis of the method of data collection and form of the data. We also compared the diagnostic performance of AI with that of experts and nonexperts. For early digestive system cancer and precancerous lesions, AI showed a high diagnostic performance in capsule endoscopy and esophageal squamous cell carcinoma. Additionally, AI-assisted endoscopic ultrasonography (EUS) had good diagnostic accuracy for pancreatic cancer. In the subgroup analysis, AI had a better diagnostic performance than experts for most digestive system tumors. However, the diagnostic performance of AI using video data requires improvement.

## 1 Introduction

The incidence and mortality rates of gastrointestinal (GI) tumors remain high. The health economic burden of these tumors is of great concern (1, 2). Early diagnosis of GI tumors is critical to achieve the best possible outcome for these patients. Endoscopy is an important method for GI tumor diagnosis, and reducing the rate of missed diagnosis is essential (3–6). Diagnosis of pancreatic tumors and mesenchymal tumors relies heavily on endoscopic ultrasonography (EUS), but the performance among EUS endoscopists varies greatly. Possible blind spots during surgery can lead to compromised patient health (7).

In recent years, the application of artificial intelligence (AI) technology (computer vision) in reducing missed diagnosis of GI tumors and improving the accuracy of EUS has received widespread attention (8, 9). However, whether the ability of AI in diagnosing all types of digestive system tumors is superior to that of experts or nonexperts is unclear (10–13). Although several meta-analyses have measured the ability of AI-assisted endoscopy to diagnose digestive system tumors, there are flaws in their study designs and the results are inconsistent. According to the largest recent surveys of endoscopists’ perceptions of AI, while most endoscopists view it positively, doubts about its diagnostic capabilities persist (14). There are fewer studies on the diagnostic capabilities of diagnostic AI in real-world clinical settings, and most are in the preclinical research stage. To avoid potential risks, a systematic evaluation of the ability of AI to aid in the diagnosis of early digestive system tumors is needed before it can be widely used in clinical practice. Therefore, we conducted a comprehensive umbrella review of this topic in the hope of contributing to the advancement of the literature in this regard.

## 2 Methods

### 2.1 Search strategy

This study was prospectively registered in PROSPERO (CRD42023445537). We strictly followed the PRIMA checklist. Institution Review Board approval and written consent are not applicable to this study. The PubMed, Web of Science, Embase, and Cochrane databases were searched to identify all (published and unpublished) meta-analyses and diagnostic studies on AI-assisted endoscopy for the diagnosis of digestive system tumors. The search was completed in July 2023. We searched the databases using a combination of Medical Subject Heading terms and keywords related to digestive system tumors, endoscopy, and AI (see **Supplementary Tables S3**, **S4** for specific search terms). Two authors (C.W.H. and Y.S.) performed separate searches to include relevant studies in the review, and any discrepancies were resolved by consultation with a third author (J.Z.D.). Additionally, meta-analyses and individual diagnostic studies were manually searched using the reference lists of all included articles.

### 2.2 Selection criteria

Meta-analyses and single diagnostic studies were eligible for inclusion if they included indicators of diagnostic performance, e.g., sensitivity and specificity. Studies were included if the outcome was CRC, pancreatic cancer, esophageal cancer, gastric cancer (GC), mesenchymal tumors, or capsule endoscopy. We extracted data on individual outcomes separately if two or more diagnostic outcomes of the disease were reported in a study. If there was more than one eligible meta-analysis of AI-assisted endoscopy for the same disease diagnostic outcome, we included the most recent study for data extraction, which was generally the study with the largest sample size (15).

The exclusion criteria for this umbrella review were articles with incorrect exposure or design (errors in data count or meta-search design and data organization), studies that did not provide any information regarding the number of patients or images, and studies published in non-English languages.

### 2.3 Data extraction

The listed authors independently extracted the following information from each eligible study: first author’s name, nationality, year of publication, tumor site, exposure factors, study design (retrospective or prospective), number of patients, and number of images (video or image data). We counted true positives (TPs), false positives (FPs), true negatives (TNs), and false negatives (FNs) for each study of AI. For articles without available TP, FP, FN, and TN data of AI, we emailed the corresponding authors of the studies to request the raw data. Studies wherein the authors did not agree to provide raw data were excluded. Additionally, we performed a grouping analysis according to the location of each tumor (capsule endoscopy, as a specific endoscopic technique, was treated as a separate group). We further sub grouped the studies according to whether the data were collected by image or video, and whether the study design was retrospective or prospective (the number of original studies included in each subgroup was more than three). We also compared the diagnoses of digestive system tumors between experts and nonexperts (experts and nonexperts were both gastroenterologists; experts were defined as having more than 5 years of experience with white light endoscopy or more than 3 years of experience with magnifying endoscopy with narrow-band imaging). It is worth noting that the images and videos involved in most of the AI models were confirmed by histopathology. For studies involving experts and nonexperts, we again extracted TPs, FPs, FNs, and TNs for experts and nonexperts. The third author (J.Z.D.) randomly extracted the data to verify the accuracy.

The AI neural network model used in most of the literature included in this paper was a convolutional neural network, which is a specific class of deep neural network that consists of convolutional and pooling layers in a pattern that resembles the organization of the visual cortex and is hence well suited for image recognition and video analysis (16). Most of the endoscopic images included were white light endoscopy and no magnified narrow-band images.

### 2.4 Statistical analysis

Pooled sensitivities and specificities with corresponding 95% confidence interval (95% CI) were calculated using a random-effects model, and forest plots (**Supplementary Figures S1**–**S7**) were constructed on the basis of these models. The Cochran Q test and I^2^ statistic were used to assess heterogeneity between the studies (17). I^2^ >50% or a p-value ≤0.1 was considered to indicate significant heterogeneity. The Egger test was used to detect potential publication bias. Statistical significance was set at a p-value <0.1 (18). Heterogeneity and publication bias were also calculated in the subgroup analysis. All statistical analyses were performed using Stata 16 (StataCorp LLC, College Station, TX, USA) and R version 4.3.1 (R Foundation for Statistical Computing, Vienna, Austria).

### 2.5 Quality assessment of the methods and evidence

The methodological quality of the meta-analyses was assessed using the AMSTAR2.0 instrument, a 16-item methodological assessment tool (19). Additionally, Grading of Recommendations, Assessment, Development, and Evaluations (GRADE) was used to assess the quality of evidence for each outcome included in the review (20). The GRADE approach categorizes evidence as “high,” “moderate,” “low,” or “very low” quality. The level of evidence can be downgraded by the risk of bias, inconsistency, indirectness, imprecision, and publication bias. The methodological quality of the studies and the quality of the evidence were independently assessed by two authors (C.W.H. and Y.S.).

## 3 Results

### 3.1 Characteristics of the meta-analysis (search, deduplication, exclusion, screening, and synthesis)

**Figure 1:**
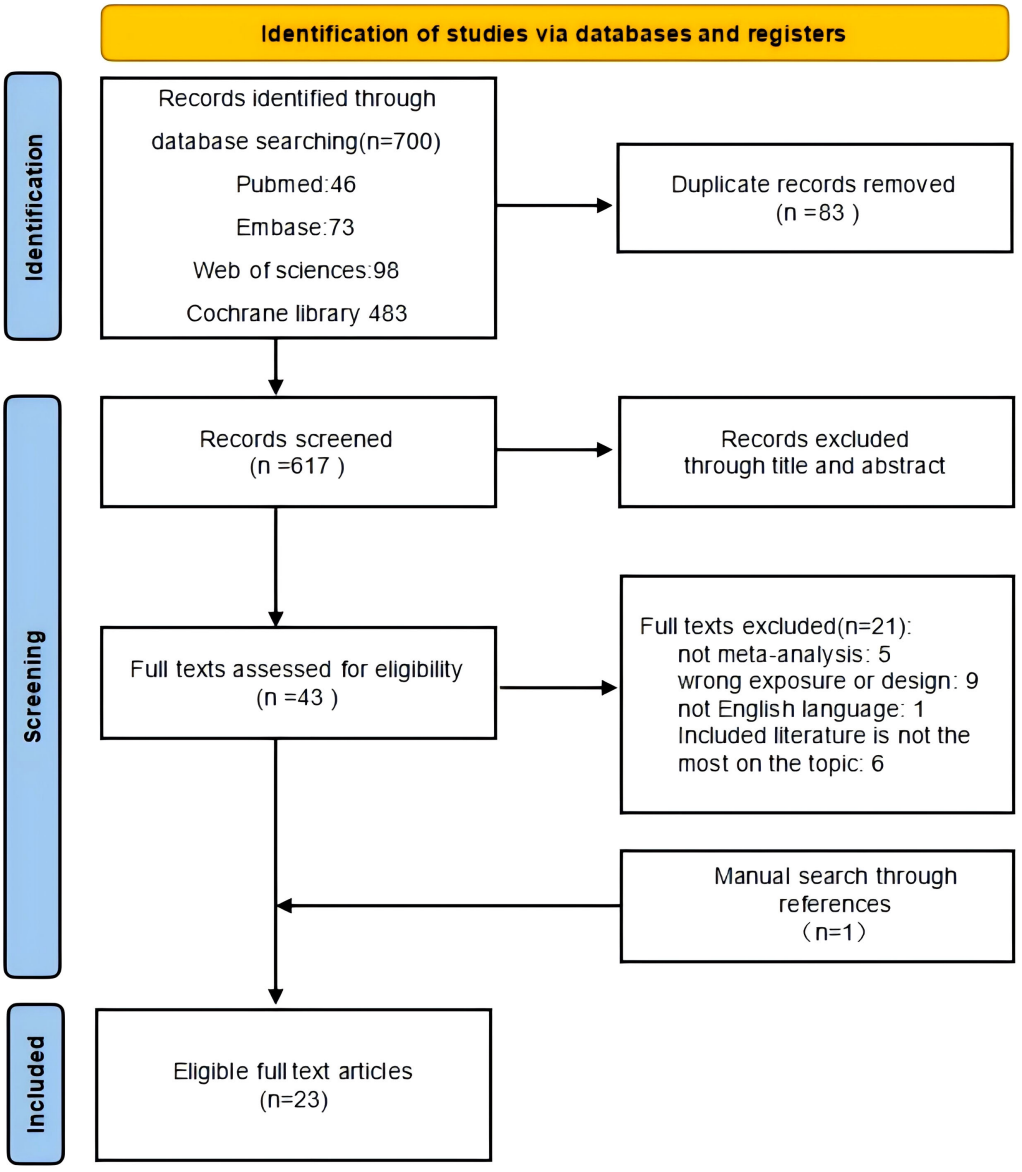
Flowchart of the systematic search and selection process.

**Figure 1** shows the flowchart of the literature search and screening. After a systematic literature search, 700 articles were identified. After screening titles and abstracts and removing duplicates, 43 articles were included. Then, 22 articles were retrieved for full-text review, of which 21 were discarded for the following reasons: one was not a meta-analysis, two studies had inappropriate designs, and three were published in languages other than English. One meta-analysis was performed by manually searching the reference lists of the included meta-analyses. Finally, 23 meta-analyses were included in this review (**Table 1**).

**Table 1:** Characteristics of the included meta-analysis.

	Number of included individual studies	GI cancer	NO. of patients	NO. of cases	Sensitivity	Speciality	Quality assessment
Babu P Mohan(2022) (12)	11	PC	2182	-	0.90 (95%CI:0.88-0.92)	0.84 (95% CI: 0.79-0.88)	Low
Elena Adriana(2022) (21)	10	PC	1871	-	0.92 (95%CI:0.89-0.95)	0.90 (95% CI: 0.83-0.94)	Very Low
Thaninee(2022) (22)	8	PC	870	-	0.91 (95%CI:0.87-0.93)	0.90 (95% CI: 0.79-0.96)	Low
Nadia Guidozzi(2023) (23)	23	EAC/ESCC	2068	-	ESCC: 0.91 (95%CI:0.84-0.95)	ESCC: 0.80 (95% CI: 0.64-0.90)	Very Low
					EAC: 0.93 (95% CI: 0.87-0.96)	EAC: 0.87 (95% CI: 0.83-0.91)	
Visaggi(2022) (24)	14	EAC/ESCC	-	-	ESCC: 0.95 (95% CI: 0.91-0.98)	ESCC: 0.92 (95% CI: 0.82-0.97)	Very Low
					EAC: 0.89 (95% CI: 0.84-0.93)	EAC: 0.86 (95% CI: 0.83-0.93)	
Julia Arribas(2020) (25)	19	EAC/ESCC/GC	1116	23878	ESCC: 0.93 (95% CI: 0.73-0.99)	ESCC: 0.89 (95% CI: 0.77-0.95)	Low
					EAC: 0.89 (95% CI: 0.83-0.93)	EAC: 0.88 (95% CI: 0.84-0.91)	
					GC: 0.88 (95% CI: 0.78-0.94)	GC: 0.89 (95% CI: 0.82-0.93)	
Islam(2022) (13)	28	EAC/ESCC	-	703006	0.938 (95% CI: 0.936-0.94)	0.917 (95% CI: 0.915-0.92)	Very Low
De Luo(2022) (10)	39	EAC/ESCC/EGC	1380	13091	EC: 0.94 (95% CI: 0.91-0.96)*/0.95 (95% CI: 0.95-0.96)	EC: 0.90 (95% CI: 0.88-0.92)*/0.95 (95% CI: 0.94-0.95)	Very Low
					0.87(95%CI:0.87-0.88)	0.88(95%CI:0.87-0.88)	
Thomas K L Lui(2020) (26)	17	EAC/ESCC/GC	-	969318	ESCC: 0.76 (95% CI: 0.48-0.93)	ESCC: 0.93 (95% CI: 0.67-0.995)	Low
					EAC: 0.88 (95% CI: 0.82-0.92)	EAC: 0.90 (95% CI: 0.86-0.95)	
					GC: 0.92 (95% CI: 0.86-0.96)	GC: 0.88 (95% CI: 0.72-0.96)	
Jin Lin Tan(2022) (27)	12	EAC	1361	532328	0.90 (95% CI: 0.87-0.93)	0.84 (95% CI: 0.80-0.88)	Low
Chang Seok Bang(2021) (28)	22	EAC/ESCC	2102	78882	0.93 (95% CI: 0.86-0.96)*/0.94 (95% CI: 0.89-0.96)	0.85 (95% CI: 0.78-0.89)*/0.88 (95% CI: 0.76-0.94)	Very Low
Pei-Chin Chen(2022) (29)	12	EGC	-	11685	0.86 (95% CI: 0.75-0.92)	0.90 (95% CI: 0.84-0.93)	Very Low
Kailin Jiang(2021) (30)	16	EGC	3787	1708519	0.86 (95% CI: 0.77-0.92)	0.93 (95% CI: 0.89-0.96)	Very Low
Islam(2021) (31)	15	EGC	7538	231096	0.89 (95% CI: 0.88-0.89)	0.89 (95% CI: 0.89-0.90)	Very Low
Xin-Yuan Liu(2022) (32)	8	GIST	533	-	0.92 (95% CI: 0.85-0.96)	0.80 (95% CI: 0.70-0.87)	Low
Jiawei Bai(2023) (33)	13	CRC	1472	13918	0.68 (95% CI: 0.59–0.76) (Japan/Korea)/0.88 (95% CI: 0.78–0.94) (China)	0.96 (95% CI: 0.93–0.98) (Japan/Korea)/0.88 (95% CI: 0.80–0.93)(China)	Low
Yixin Xu(2021) (11)	13	CRC	-	234266	0.85 (95% CI: 0.69–0.93)	0.97 (95% CI: 0.95–0.98)	Very Low
Aling Wang(2021) (34)	26	CRC	-	5246543	0.88 (95% CI: 0.81-0.92)	0.95 (95% CI: 0.94–0.96)	Low
Thomas K L Lui(2020) (35)	18	CRC	-	7680	0.92 (95% CI: 0.89-0.95)	0.90 (95% CI: 0.85-0.93)	Low
Ming-De Li(2022) (36)	16	CRC	-	33388	0.93 (95% CI: 0.91-0.95)	0.87 (95% CI: 0.76-0.93)	Very Low
Chang Seok Bang(2021) (37)	13	CRC	-	6564	0.88 (95% CI: 0.87–0.88)	0.79 (95% CI: 0.78–0.80)	Low
Junjie Mi(2022) (38)	8	CRC (WCE)	819	18414	0.97 (95% CI: 0.95-0.98)	0.97 (95% CI: 0.94–0.98)	Very Low
Hye Jin Kim(2022) (39)	7	Intestinal polyp (WCE)	-	28148	0.97 (95% CI: 0.82-0.99)	0.98 (95% CI: 0.92-0.99)	Low

PC, pancreatic cancer; EAC, esophageal adenocarcinoma; ESCC, esophageal squamous cell carcinoma; EGC, early gastric cancer; GIST, gastrointestinal stromal tumors; CRC, colorectal cancer; WCE, wireless capsule endoscopy.

*patients #cases.

### 3.2 Characteristics of the data

The studies reported on AI-assisted endoscopic diagnosis of pancreatic (n=3), esophageal (n=8), gastric (n=6), and colorectal (n=6) cancers; mesenchymal tumors (n=1); and AI-assisted capsule endoscopy for the diagnosis of GI tumors (n=2). Our literature search for individual diagnostic studies not included in the published meta-analyses identified 25 additional studies (the original studies had the same inclusion and exclusion criteria as the meta-analysis): three studies on the diagnosis of pancreatic cancer, two studies on the diagnosis of esophageal cancer, 17 studies on the diagnosis of gastric cancer (GC), and three studies on the diagnosis of mesenchymal tumors. After removing duplicates, 193 original articles were included. We compared AI’s performance with the gold standard pathological diagnosis as follows: TP, correctly diagnosed patients with tumors; TN, correctly diagnosed healthy individuals; FP, incorrectly diagnosed healthy individuals as having tumors; and FN, incorrectly diagnosed individuals with tumors as healthy. Sensitivity (TP/TP+FN) reflects the ability of AI to detect patients, with higher sensitivity indicating fewer missed diagnoses. Meanwhile, specificity (TN/TN+FP) reflects the ability to correctly identify patients without the condition; the higher the specificity, the lower the misdiagnosis rate (10).

### 3.3 Quality assessment of the meta-analyses

The quality of the included meta-analyses was assessed using AMSTAR (version 2). **Supplementary Table S1** presents details of the quality assessment of the 23 included meta-analyses. There was no “high” or “moderate” quality evidence. Eleven of the studies were of “low” quality, and 12 of the studies were of “very low” quality.

### 3.4 Heterogeneity

**Figure 2:**
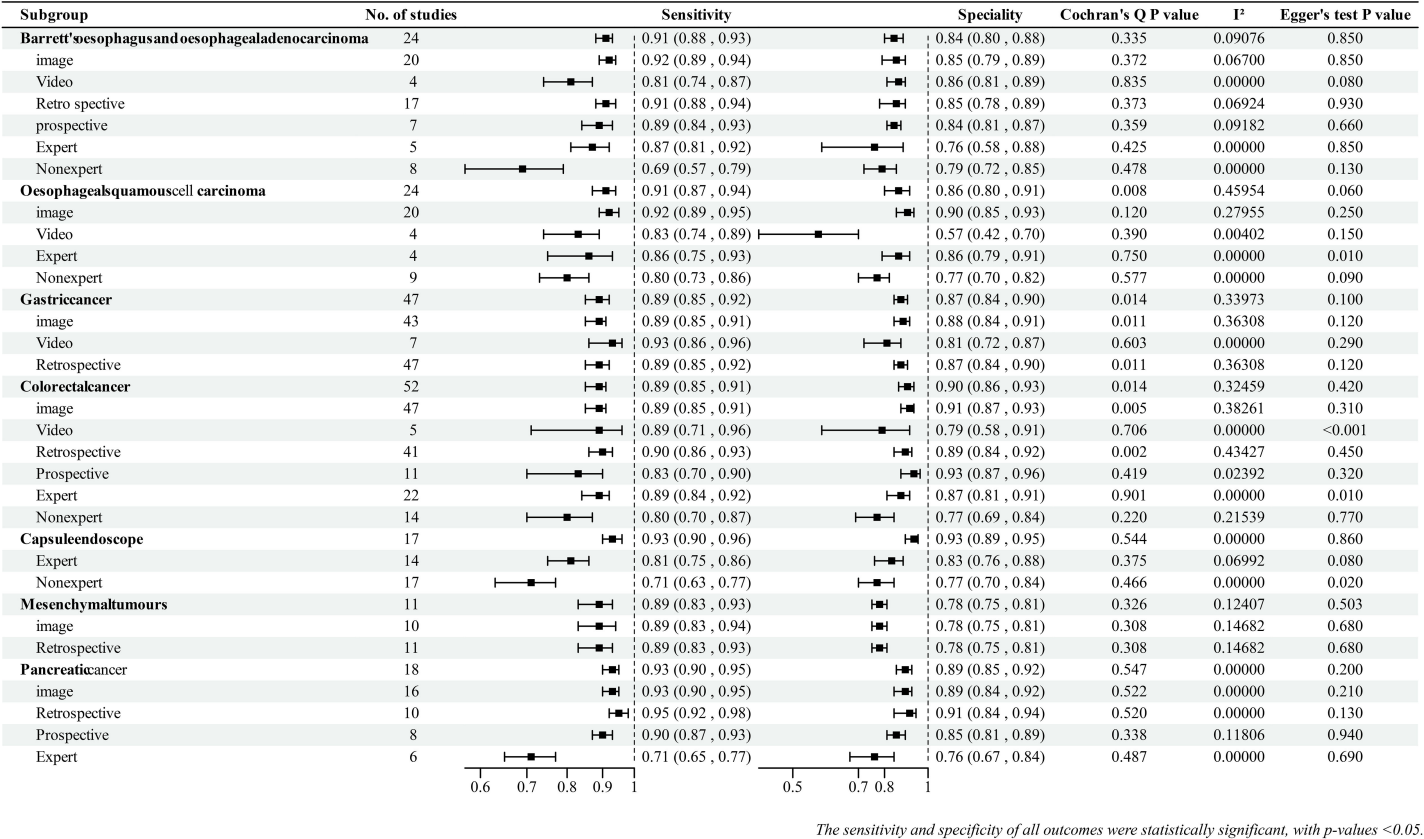
Summary findings for each outcome.

The I^2^ statistic and Cochran Q test were used to detect possible heterogeneity between the studies. Seven (22%) outcomes had significant heterogeneity (I^2^ >50% or p-value ≤0.1), and the rest (78%) had no significant heterogeneity (**Figure 2**). The main reasons for heterogeneity were differences in AI methods and endoscopic imaging techniques, differences in quality and quantity of endoscopic images and videos, and differences in study design. Additionally, the reasons for greater heterogeneity in the image subgroup compared with the video subgroup and in the retrospective subgroup compared with the prospective subgroup were the large number of included studies and differences in AI algorithms and imaging techniques.

### 3.5 Group and subgroup

#### 3.5.1 GI tumors

The abilities of AI-assisted endoscopy to diagnose esophageal tumors (Barrett esophagus and esophageal adenocarcinoma, and esophageal squamous cell carcinoma), GC, and colorectal cancers (CRCs), and AI-assisted capsule endoscopy to diagnose GI tumors are summarized as follows. Details of the summary effect sizes are shown in **Figure 2**.

In terms of tumor location, AI-assisted capsule endoscopy showed excellent performance in diagnosing GI tumors with a pooled sensitivity of 0.93 (95% CI: 0.90–0.96) and pooled specificity of 0.93 (95% CI: 0.89–0.95).In gastroenteroscopy, AI-assisted endoscopy exhibited the best diagnostic performance for esophageal squamous cell carcinoma, followed by Barrett esophagus, esophageal adenocarcinoma, colorectal cancer, and GC.

Most groups showed better diagnostic performance with image data than with video data. This was observed in the groups of esophageal adenocarcinoma [EAC], pooled specificity: 0.85 [95% CI: 0.79–0.89]), esophageal squamous cell carcinoma [ESCC], and CRC. The GC group showed similar performance in the picture and video subgroups.

For prospective studies, no data existed on ESCC, and the available prospective studies for GC were limited. Except for the CRC group, the diagnostic performance of AI in retrospective and prospective studies was not significantly different; however, AI performed better in retrospective studies than in prospective studies.

We compared the diagnostic performance of AI with that of experts and nonexperts (expert and nonexpert diagnostic capabilities were meta-analyzed on the basis of the extracted data).he combined results showed that most AI models had better diagnostic capabilities than experts (Summary effect sizes are shown in **Figure 2**). Interestingly, three studies (40–42) analyzed the ability of nonexperts in diagnosing GC under endoscopy; with the help of AI, the diagnostic performance of nonexperts was found to reach the level of experts. Two studies (43, 44) reported that AI-assisted nonexperts achieved diagnostic performance comparable to that of experts in CRC.

#### 3.5.2 Pancreatic tumors and mesenchymal tumors

Studies on AI-assisted EUS for the diagnosis of digestive system tumors are fewer, focusing on pancreatic tumors and mesenchymal tumors. Overall, the diagnostic performance of AI for pancreatic tumors was superior to that of mesenchymal tumors. Similar to GI tumors, the diagnostic performance of AI-assisted EUS was better than that of experts. There were no significant differences between retrospective and prospective studies.

### 3.6 Assessment of the risk of bias

Publication bias was found for ESCC (expert and nonexpert subgroups), Barrett esophagus and EAC (video subgroup), CRC (video and expert subgroups), and GC (expert and nonexpert subgroups). The remaining outcomes did not exhibit significant publication bias.

### 3.7 Grade

We downgraded the evidence according to five factors (risk of bias, inconsistency, indirectness, imprecision, and publication bias). The evidence for the following outcomes was downgraded to “moderate” quality: GC group, GC (image subgroup, retrospective subgroup, expert subgroup, and nonexpert subgroup), CRC group, CRC (image subgroup, video subgroup, retrospective subgroup, and expert subgroup), Barrett esophagus and EAC (video subgroup), and ESCC (expert subgroup and nonexpert subgroup). Only one piece of evidence was downgraded to “low” quality (ESCC group). The primary reasons for this downgrading were inconsistencies and publication bias. The evidence for the remaining outcomes was of “high” quality (**Supplementary Table S2**).

## 4 Discussion

Our results showed that the use of AI improved the detection and diagnostic accuracy of early digestive system tumors. Furthermore, AI-assisted endoscopic ultrasonography (EUS) had good diagnostic accuracy for pancreatic cancer.; meanwhile, AI showed high diagnostic performance in capsule endoscopy and esophageal squamous cell carcinoma for early digestive system tumors and precancerous lesions. Additionally, we compared the diagnostic capabilities of AI with those of experts and found that the diagnostic capability of AI was superior to that of experts.

During endoscopy, endoscopists must obtain diagnostic information while performing the endoscopy; therefore, most real-life scenarios are similar to video data formats. Therefore, we subdivided the AI into image subgroups and video subgroups. In this study, most subgroups using video data had lower diagnostic capability than those using image data. Therefore, we concluded that, although AI has a high diagnostic accuracy using image data, its diagnostic capability may be limited during clinical endoscopic procedures.

Prospective studies have a higher level of evidence compared to other studies. We performed meta-analysis on prospective studies of esophageal adenocarcinoma, colorectal cancer, and pancreatic cancer. The results showed that AI’s diagnostic performance in the prospective study group surpassed that of the experts and was higher than video-based diagnostic performance. This supports our conclusion that AI’s diagnostic capability surpasses that of experts, though AI’s performance with video data still requires improvement. Notably, although fewer prospective studies were available for gastric cancer in our review, the results from these studies also align with the conclusion mentioned above. Regarding the outcome of CRC, we found that the retrospective subgroup had higher sensitivity, whereas the prospective subgroup had higher specificity. However, this was not the case in other retrospective and prospective subgroups. We believe that a possible reason for this result is that there may have been selection bias when retrospectively collecting CRC data, which may lead researchers to select images with better bowel preparation scores, resulting in higher sensitivity. Overall, AI’s diagnostic capabilities in prospective studies require further improvement when compared to retrospective studies. This indicates a need for additional prospective studies to validate AI’s diagnostic abilities.

Overall, there was less heterogeneity in the outcomes, except for esophageal squamous carcinoma, GC, and CRC. Regarding the risk of bias, there was greater publication bias associated with esophageal cancer and CRC and almost no publication bias in the remaining outcomes. Selection bias in retrospective studies is inevitable. Many retrospective studies were included in this study; therefore, the selection bias was high. Regarding the evidence rating, the performance of AI in diagnosing ESCC was low-level evidence, whereas those of the remaining studies were medium- and high-level evidences. Although a small number of randomized controlled trials have evaluated the ability of AI to assist in the early diagnosis of cancer in clinical settings, most applications of AI for the endoscopic diagnosis of digestive system tumors are still in the preliminary stage (45–47).

AI is not yet widely used in clinical practice; thus, our study systematically summarizes the current research on the diagnostic capability of AI-assisted diagnosis of digestive system tumors and presents a pooled analysis of all data containing comparisons with experts and nonexperts. This study provides the latest evidence of AI-assisted endoscopy for the diagnosis of early digestive system tumors and precancerous lesions. The results of this study have practical implications in guiding the development of real-world applications.

In the past few years, the application of AI to endoscopic clinical practice has received increasing attention. AI can recognize subtle changes that cannot be identified by traditional methods and help identify subtle lesions. During endoscopy, AI can match multiple endoscopic imaging modalities, such as white light endoscopy (WEL) and narrowband imaging (NBI). AI can also help to label suspicious lesions in real time (12, 14).Thus, AI has been shown to improve the detection rate of digestive tumors, especially for less experienced endoscopists. AI-assisted novice endoscopists have lower rates of missed diagnoses, with results not inferior to the expert level (48). Interestingly, AI can also assign monitoring intervals to patients after polypectomy (49), and it can even improve bowel preparation (50). In recent years, automated endoscopic reporting systems have received increasing attention. One study (51) showed that the use of an AI-based endoscopy automatic reporting system significantly improved the accuracy and completeness of esophagogastroduodenoscopy (EGD) reporting, reduced the work burden of endoscopists, and promises to be an enhanced tool for EGD recording services. However, before focusing on the diagnostic capabilities of automated endoscopic reporting systems, it is essential to summarize the diagnostic capabilities of AI. Other prospective studies assessing AI’s performance in diagnosing digestive tumors have yielded important results. Most of these studies reported results aligning with our findings that the application of AI can improve the diagnosis rate of digestive system tumors (52–56). However, some prospective studies have indicated that AI may not enhance the diagnosis rate of these tumors (57, 58). We speculate that this discrepancy may stem from the fact that studies reporting no improvement were primarily single-center studies. One study involved a small sample size, and the other included a high-risk oncology hospital population, potentially influencing the results.

Based on previous meta-analysis and diagnostic tests (36, 41), we defined physicians with 5 years of experience as endoscopists. Although our study provided a uniform definition of “expert,” some diagnostic experiments use more detailed definitions, such as the number of operational cases or recognition by an academic association. This leads to heterogeneity in the definition of “experts.” The AI models or algorithms also differ between studies. These differences will have an unpredictable influence on the research results. Additionally, the prospective and video subgroups included a smaller number of studies, which also affected the results. Therefore, further prospective studies on the diagnostic capabilities of AI models based on video data are required to provide the latest evidence.

Still, before AI can be used in large-scale clinical applications, several problems must be resolved. First, the establishment of AI models requires large amounts of patient data. Owing to the lack of policies regarding the use of training data, there is a substantial risk of patient information leakage, and formulating relevant regulations is recommended to avoid potential risks (59). Additionally, to gain the trust of doctors and patients in the clinical stage of AI application, AI is required to achieve better diagnostic capability and interpretability. Our current research provides the latest evidence for the diagnostic capability of AI in the endoscopic diagnosis of digestive system tumors, indicating that AI has an excellent diagnostic performance; yet, convincing patients of the diagnosis of AI requires more popular science (60, 61).

Although there have been some active attempts to address the “black box” problem in neural networks, we cannot adequately explain the results produced by current AI (62). Most importantly, whether the responsibility for the errors that occur when AI is used in clinical applications lies with the endoscopic technologist, AI developer, or regulator cannot be answered (63–65).

Despite the good diagnostic performance achieved by AI, there are still some problems to be solved before its large-scale clinical application. Sound policies and regulations need to be developed to address the ethical issues associated with AI applications.

## 5 Conclusion

This is an umbrella review to evaluate the diagnostic performance of artificial intelligence (AI)-assisted endoscopy for digestive tumors. The results indicate that, for early digestive system cancer and precancerous lesions, AI showed a high diagnostic performance in capsule endoscopy and esophageal squamous cell carcinoma. Additionally, AI-assisted endoscopic ultrasonography (EUS) had good diagnostic accuracy for pancreatic cancer. In the subgroup analysis, AI had a better diagnostic performance than experts for most digestive system tumors. However, the diagnostic performance of AI using video data requires improvement.

## Data Availability

The original contributions presented in the study are included in the article/**Supplementary Material**. Further inquiries can be directed to the corresponding author/s.
