# Supplementary material for: Diagnostic performance of AI-assisted endoscopy diagnosis of digestive system tumors: an umbrella review
Source: Front Oncol. 2025 Apr 3;15:1519144. doi: 10.3389/fonc.2025.1519144 (PMC12003149; doi:10.3389/fonc.2025.1519144)
Supplement: Supplementary file 1 [file DataSheet1.doc]

**Supplementary material**

**Table S 1** AMSTAR-2

**Table S 2** GRADE

**Table S 3** Search strategy for the Meta-analysis in PubMed, EMBASE,Web of Science and Cochrane

**Table S 4** Search strategy for individual diagnostic studies in PubMed, EMBASE,Web of Science and Cochrane

**Figure S 1** Pooled sensitivity and specificity of AI-assisted endoscopy in the diagnosis of Pancreatic cancer

**Figure S 2** Pooled sensitivity and specificity of AI-assisted endoscopy in the diagnosis of Barrett's esophageal adenocarcinoma

**Figure S 3** Pooled sensitivity and specificity of AI-assisted endoscopy in the diagnosis of Esophageal squamous cell carcinoma

**Figure S 4** Pooled sensitivity and specificity of AI-assisted endoscopy in the diagnosis of Gastric cancer

**Figure S 5** Pooled sensitivity and specificity of AI-assisted endoscopy in the diagnosis of mesenchymal stromal tumor

**Figure S 6** Pooled sensitivity and specificity of AI-assisted endoscopy in the diagnosis of Colorectal cancer

**Figure S 7** AI-assisted WCE in the diagnosis of gastrointestinal tumors

**Table S 1** Detailed evaluation of the methodological quality with AMSTAR 2.

| Author,year | 1 | 2 | 3 | 4 | 5 | 6 | 7 | 8 | 9 | 10 | 11 | 12 | 13 | 14 | 15 | 16 |  |  |
| --- | --- | --- | --- | --- | --- | --- | --- | --- | --- | --- | --- | --- | --- | --- | --- | --- | --- | --- |
| **Pancreatic cancer** |  |  |  |  |  |  |  |  |  |  |  |  |  |  |  |  |  |  |
| [Babu P Mohan](https://pubmed.ncbi.nlm.nih.gov/?term=Mohan+BP&cauthor_id=35313417),2022 | N | Y | N | N | N/A | Y | N | PY | Y | N | Y | Y | Y | Y | Y | Y | Low |  |
| [Elena Adriana Dumitrescu](https://pubmed.ncbi.nlm.nih.gov/?term=Dumitrescu+EA&cauthor_id=35204400),2022 | N | Y | N | N | Y | Y | N | PY | Y | N | Y | Y | Y | Y | N | Y |  | Very low |
| [Thaninee Prasoppokakorn](https://pubmed.ncbi.nlm.nih.gov/?term=Prasoppokakorn+T&cauthor_id=34937308),2022 | N | Y | N | N | Y | Y | N | PY | Y | N | Y | Y | Y | Y | Y | Y | Low |  |
| **Esophagus cancer and Gastric cancer** |  |  |  |  |  |  |  |  |  |  |  |  |  |  |  |  |  |  |
| [Nadia Guidozzi](https://pubmed.ncbi.nlm.nih.gov/?term=Guidozzi+N&cauthor_id=37480192),2023 | N | N | N | N | N/A | N/A | N | PY | N | N | Y | Y | Y | Y | N | N |  | Very low |
| [Pierfrancesco Visaggi](https://pubmed.ncbi.nlm.nih.gov/?term=Visaggi+P&cauthor_id=35098562),2022 | N | N | N | N | Y | Y | N | PY | Y | N | Y | Y | Y | Y | Y | Y |  | Very low |
| [Julia Arribas](https://pubmed.ncbi.nlm.nih.gov/?term=Arribas+J&cauthor_id=33127833),2020 | N | Y | N | N | Y | Y | N | PY | Y | N | Y | Y | Y | Y | Y | Y | Low |  |
| [Md Mohaimenul Islam](https://pubmed.ncbi.nlm.nih.gov/?term=Islam+MM&cauthor_id=36497480),2022 | N | N | N | N | Y | Y | N/A | PY | Y | N | Y | Y | Y | Y | Y | Y |  | Very low |
| [De Luo](https://pubmed.ncbi.nlm.nih.gov/?term=Luo+D&cauthor_id=35756602),2022 | N | Y | N | N | Y | Y | N | PY | Y | N | Y | Y | Y | N/A | N | Y |  | Very low |
| [Thomas K L Lui](https://pubmed.ncbi.nlm.nih.gov/?term=Lui+TKL&cauthor_id=32562608),2020 | N | Y | N | N | Y | N/A | N | PY | Y | N | Y | Y | Y | Y | Y | N/A | Low |  |
| [Jin Lin Tan](https://pubmed.ncbi.nlm.nih.gov/?term=Tan+JL&cauthor_id=35814747),2022 | Y | Y | N | N | Y | Y | PN | PY | Y | N | Y | Y | Y | Y | Y | Y | Low |  |
| [Chang Seok Bang](https://pubmed.ncbi.nlm.nih.gov/?term=Bang+CS&cauthor_id=33290771),2021 | N | Y | N | N | Y | Y | PN | Y | Y | N | Y | Y | Y | Y | Y | Y |  | Very low |
| [Pei-Chin Chen](https://pubmed.ncbi.nlm.nih.gov/?term=Chen+PC&cauthor_id=35576561),2022 | N | Y | N | N | Y | Y | N | PY | Y | N | Y | Y | Y | Y | N | Y |  | Very low |
| [Kailin Jiang](https://pubmed.ncbi.nlm.nih.gov/?term=Jiang K[Author]),2021 | N | N | N | N | N/A | Y | N | PY | Y | N | Y | Y | Y | Y | Y | Y |  | Very low |
| [Md Mohaimenul Islam](https://pubmed.ncbi.nlm.nih.gov/?term=Islam+MM&cauthor_id=34771416),2021 | N | N | N | N |  |  |  |  |  |  |  |  |  |  |  |  |  | Very low |
| **Gastrointestinal stromol tumor** |  |  |  |  |  |  |  |  |  |  |  |  |  |  |  |  |  |  |
| [Xin-Yuan Liu](https://pubmed.ncbi.nlm.nih.gov/?term=Liu XY[Author]),2022 | N | Y | N | PN | Y | Y | PN | PY | Y | N | Y | Y | Y | Y | Y | Y | Low |  |
| **Colorectal cancer** |  |  |  |  |  |  |  |  |  |  |  |  |  |  |  |  |  |  |
| [Jiawei Bai](https://pubmed.ncbi.nlm.nih.gov/?term=Bai+J&cauthor_id=37430125),2023 | N | Y | N | PN | N/A | Y | PN | PY | Y | Y | Y | Y | Y | Y | Y | Y | Low |  |
| [Yixin Xu](https://pubmed.ncbi.nlm.nih.gov/?term=Xu Y[Author]),2021 | N | N | N | PN | N/A | Y | PN | PY | Y | Y | Y | Y | Y | Y | Y | Y |  | Very low |
| [Aling Wang](https://pubmed.ncbi.nlm.nih.gov/?term=Wang A[Author]),2021 | N | Y | N | PN | N/A | Y | PN | PY | Y | Y | Y | Y | Y | Y | Y | Y | Low |  |
| [Thomas K L Lui](https://pubmed.ncbi.nlm.nih.gov/?term=Lui+TKL&cauthor_id=32119938),2020 | N | Y | N | PN | Y | Y | PN | PY | Y | Y | Y | Y | Y | Y | Y | Y | Low |  |
| [Ming-De Li](https://pubmed.ncbi.nlm.nih.gov/?term=Li MD[Author]),2022 | N | N |  |  |  |  |  |  |  |  |  |  |  |  |  |  |  | Very low |
| [Chang Seok Bang](https://pubmed.ncbi.nlm.nih.gov/?term=Bang CS[Author]),2021 | N | Y |  | PN |  |  | PN |  |  |  |  |  |  |  |  |  | Low |  |
| **Capsule endoscope** |  |  |  |  |  |  |  |  |  |  |  |  |  |  |  |  |  |  |
| [Junjie Mi](https://pubmed.ncbi.nlm.nih.gov/?term=Mi J[Author]),2022 | N | N | N | PN | Y | Y | PN | PY | Y | Y | Y | Y | Y | Y | Y | Y |  | Very low |
| [Hye Jin Kim](https://pubmed.ncbi.nlm.nih.gov/?term=Kim HJ[Author]),2022 | N | Y | N | PN | Y | Y | PN | PY | Y | Y | Y | Y | Y | Y | Y | Y | Low |  |

**Abbreviation:** Y yes; N no; PY partial yes;PN partial no; N/A not applicable due to absence of meta-analyses. 1 PICO elements; 2 prior protocol; 3 study designs; 4 search strategy; 5 study selection; 6 data extraction; 7 excluded studies; 8 PIC details; 9 risk of bias assessment; 10 funding sources; 11 meta-analysis methods; 12 risk of bias impact on results; 13 risk of bias discussion; 14 explain heterogeneity; 15 publication bias; 16 conflict of interest

**Table S 2** GRADE

| Outcome | Subgroup | Risk of bias | Inconsistency | Inderectness | Imprecision | Publication bias | Overall certainty of evidence |
| --- | --- | --- | --- | --- | --- | --- | --- |
| Pancreatic cancer | ✱ | NS | NS | NS | NS | NS | High |
|  | image | NS | NS | NS | NS | NS | High |
|  | Retrospective | NS | NS | NS | NS | NS | High |
|  | Prospective | NS | NS | NS | NS | NS | High |
| Barrett's esophageal adenocarcinoma | ✱ | NS | NS | NS | NS | NS | High |
|  | image | NS | NS | NS | NS | NS | High |
|  | Video | NS | NS | NS | NS | S | Moderate |
|  | Retrospective | NS | NS | NS | NS | NS | High |
|  | prospective | NS | NS | NS | NS | NS | High |
|  | Expert | NS | NS | NS | NS | NS | High |
|  | Nonexprt | NS | NS | NS | NS | NS | High |
| Esophageal squamous cell carcinoma | ✱ | NS | NS | S | NS | S | Low |
|  | image | NS | NS | NS | NS | NS | High |
|  | Video | NS | NS | NS | NS | NS | High |
|  | Expert | NS | NS | NS | NS | S | Moderate |
|  | Nonexpert | NS | NS | NS | NS | S | Moderate |
| Gastric cancer | ✱ | NS | NS | S | NS | NS | Moderate |
|  | image | NS | NS | S | NS | NS | Moderate |
|  | Video | NS | NS | NS | NS | NS | High |
|  | Retrospective | NS | NS | S | NS | NS | Moderate |
|  | Expert | NS | NS | NS | NS | S | Moderate |
|  | Nonexpert | NS | NS | NS | NS | S | Moderate |
| mesenchymal stromal tumor | ✱ | NS | NS | NS | NS | NS | High |
|  | image | NS | NS | NS | NS | NS | High |
|  | Retrospective | NS | NS | NS | NS | NS | High |
|  | Expert | NS | NS | NS | NS | NS | High |
| Colorectal cancer | ✱ | NS | NS | S | NS | NS | Moderate |
|  | image | NS | NS | S | NS | NS | Moderate |
|  | Video | NS | NS | NS | NS | S | Moderate |
|  | Retrospective | NS | NS | S | NS | NS | Moderate |
|  | Prospective | NS | NS | NS | NS | NS | High |
|  | Expert | NS | NS | NS | NS | S | Moderate |
|  | Nonexpert | NS | NS | NS | NS | NS | High |
| Capsule endoscope | ✱ | NS | NS | NS | NS | NS | High |

*:all studies(group); NS not serious; S serious

**Table S 3** Search strategy for the Meta-analysis in PubMed, EMBASE,Web of Science and Cochrane

| Database | Search strategy |
| --- | --- |
| PUBMED | ("artificial intelligence"[MeSH Terms] OR ("AI"[Title/Abstract] OR "intelligence artificial"[Title/Abstract] OR "computational intelligence"[Title/Abstract] OR "intelligence computational"[Title/Abstract] OR "machine intelligence"[Title/Abstract] OR "intelligence machine"[Title/Abstract] OR "computer reasoning"[Title/Abstract] OR "reasoning computer"[Title/Abstract] OR "computer vision systems"[Title/Abstract] OR "computer vision system"[Title/Abstract] OR "system computer vision"[Title/Abstract] OR "systems computer vision"[Title/Abstract] OR (("vision s"[All Fields] OR "vision, ocular"[MeSH Terms] OR ("Vision"[All Fields] AND "ocular"[All Fields]) OR "ocular vision"[All Fields] OR "Vision"[All Fields] OR "visions"[All Fields] OR "visioning"[All Fields]) AND "system computer"[Title/Abstract]) OR (("education"[MeSH Terms] OR "education"[All Fields] OR ("knowledge"[All Fields] AND "acquisition"[All Fields]) OR "knowledge acquisition"[All Fields]) AND "Computer"[Title/Abstract]) OR ((("acquisition"[All Fields] OR "acquisitions"[All Fields]) AND ("knowledge"[MeSH Terms] OR "knowledge"[All Fields] OR "knowledge s"[All Fields] OR "knowledgeability"[All Fields] OR "knowledgeable"[All Fields] OR "knowledgeably"[All Fields] OR "knowledges"[All Fields])) AND "Computer"[Title/Abstract]) OR ((("knowledge"[MeSH Terms] OR "knowledge"[All Fields] OR "knowledge s"[All Fields] OR "knowledgeability"[All Fields] OR "knowledgeable"[All Fields] OR "knowledgeably"[All Fields] OR "knowledges"[All Fields]) AND ("representability"[All Fields] OR "representable"[All Fields] OR "representation"[All Fields] OR "representation s"[All Fields] OR "representational"[All Fields] OR "representations"[All Fields])) AND "Computer"[Title/Abstract]) OR ((("knowledge"[MeSH Terms] OR "knowledge"[All Fields] OR "knowledge s"[All Fields] OR "knowledgeability"[All Fields] OR "knowledgeable"[All Fields] OR "knowledgeably"[All Fields] OR "knowledges"[All Fields]) AND ("representability"[All Fields] OR "representable"[All Fields] OR "representation"[All Fields] OR "representation s"[All Fields] OR "representational"[All Fields] OR "representations"[All Fields])) AND "Computer"[Title/Abstract]) OR ((("representability"[All Fields] OR "representable"[All Fields] OR "representation"[All Fields] OR "representation s"[All Fields] OR "representational"[All Fields] OR "representations"[All Fields]) AND ("knowledge"[MeSH Terms] OR "knowledge"[All Fields] OR "knowledge s"[All Fields] OR "knowledgeability"[All Fields] OR "knowledgeable"[All Fields] OR "knowledgeably"[All Fields] OR "knowledges"[All Fields])) AND "Computer"[Title/Abstract]) OR "machine learning"[Title/Abstract] OR "deep learning"[Title/Abstract] OR "supervised machine learning"[Title/Abstract] OR "support vector machine"[Title/Abstract] OR "unsupervised machine learning"[Title/Abstract] OR "diagnosis computer assisted"[Title/Abstract] OR "image interpretation computer assisted"[Title/Abstract])) AND ("endoscopes"[MeSH Terms] OR ("Colonoscopy"[Title/Abstract] OR "Endoscopy"[Title/Abstract] OR "Gastroscopy"[Title/Abstract] OR "Sigmoidoscopy"[Title/Abstract] OR "Endoscopy"[Title/Abstract] OR "EGDS"[Title/Abstract] OR "esofagogastroduodenoscopy"[Title/Abstract] OR "Colonoscopy"[Title/Abstract] OR "Sigmoidoscopy"[Title/Abstract] OR "proctoscopy"[Title/Abstract] OR "EUS"[Title/Abstract] OR "Endosonography"[Title/Abstract] OR ("endoscope"[Title/Abstract] AND "ultrasound"[Title/Abstract]))) AND ("meta analysis"[Publication Type] OR "meta analysis as topic"[MeSH Terms] OR "meta analysis"[All Fields] OR "Meta-analytic"[All Fields]) AND ("Neoplasms"[MeSH Terms] OR ("Tumor"[Title/Abstract] OR "Neoplasm"[Title/Abstract] OR "Tumors"[Title/Abstract] OR "Neoplasia"[Title/Abstract] OR "Neoplasias"[Title/Abstract] OR "Cancer"[Title/Abstract] OR "Cancers"[Title/Abstract] OR "malignant neoplasm"[Title/Abstract] OR "Malignancy"[Title/Abstract] OR "Malignancies"[Title/Abstract] OR "malignant neoplasms"[Title/Abstract] OR "neoplasm malignant"[Title/Abstract] OR "neoplasms malignant"[Title/Abstract] OR "benign neoplasms"[Title/Abstract] OR "benign neoplasm"[Title/Abstract] OR "neoplasms benign"[Title/Abstract] OR "neoplasm benign"[Title/Abstract]) OR "adenocarcinoma"[MeSH Terms] OR ("adenoma malignant"[Title/Abstract] OR "adenomas malignant"[Title/Abstract] OR "malignant adenoma"[Title/Abstract]) OR "pancreas"[MeSH Terms] OR ("Pancrea"[Title/Abstract] OR "pancreas"[Title/Abstract]) OR ("Stomach"[MeSH Terms] OR "Stomach"[Title/Abstract] OR "gastric"[Title/Abstract]) OR ("digestive system"[MeSH Terms] OR "digestive system"[Title/Abstract]) OR ("gastrointestinal tract"[MeSH Terms] OR "gastrointestinal tract"[Title/Abstract]) OR ("colon"[MeSH Terms] OR "colon"[Title/Abstract] OR "rectum"[Title/Abstract] OR "colorectum"[Title/Abstract] OR "Colorectal"[Title/Abstract]) OR ("colorectal neoplasms"[MeSH Terms] OR "colorectal neoplasms"[Title/Abstract]) OR ("adenomatous polyposis coli"[MeSH Terms] OR "adenomatous polyposis coli"[Title/Abstract]) OR ("Esophagus"[MeSH Terms] OR "Esophagus"[Title/Abstract]) OR ("gastrointestinal stromal tumor"[Title/Abstract] OR "gastrointestinal stromal tumors"[Title/Abstract] OR "GIST"[Title/Abstract] OR "GISTS"[Title/Abstract])) |
| EMBASE | (('artificial intelligence':ab,ti OR ('AI':ab,ti OR 'intelligence artificial':ab,ti OR 'computational intelligence':ab,ti OR 'intelligence computational':ab,ti OR 'machine intelligence':ab,ti OR 'intelligence machine':ab,ti OR 'computer reasoning':ab,ti OR 'reasoning computer':ab,ti OR 'computer vision systems':ab,ti OR 'computer vision system':ab,ti OR 'system computer vision':ab,ti OR 'systems computer vision':ab,ti OR (('vision s':ab,ti OR 'vision, ocular':ab,ti OR ('Vision':ab,ti AND 'ocular':ab,ti) OR 'ocular vision':ab,ti OR 'Vision':ab,ti OR 'visions':ab,ti OR 'visioning':ab,ti) AND 'system computer':ab,ti) OR (('education':ab,ti OR ('knowledge':ab,ti AND 'acquisition':ab,ti) OR 'knowledge acquisition':ab,ti) AND 'Computer':ab,ti) OR ((('acquisition':ab,ti OR 'acquisitions':ab,ti) AND ('knowledge':ab,ti OR 'knowledge':ab,ti OR 'knowledges':ab,ti OR 'knowledgeability':ab,ti OR 'knowledgeable':ab,ti OR 'knowledgeably':ab,ti OR 'knowledges':ab,ti)) AND 'Computer':ab,ti) OR ((('knowledge':ab,ti OR 'knowledge':ab,ti OR 'knowledge s':ab,ti OR 'knowledgeability':ab,ti OR 'knowledgeable':ab,ti OR 'knowledgeably':ab,ti OR 'knowledges':ab,ti) AND ('representability':ab,ti OR 'representable':ab,ti OR 'representation':ab,ti OR 'representation s':ab,ti OR 'representational':ab,ti OR 'representations':ab,ti)) AND 'Computer':ab,ti) OR ((('knowledge':ab,ti OR 'knowledge':ab,ti OR 'knowledge s':ab,ti OR 'knowledgeability':ab,ti OR 'knowledgeable':ab,ti OR 'knowledgeably':ab,ti OR 'knowledges':ab,ti) AND ('representability':ab,ti OR 'representable':ab,ti OR 'representation':ab,ti OR 'representation s':ab,ti OR 'representational':ab,ti OR 'representations':ab,ti)) AND 'Computer':ab,ti) OR ((('representability':ab,ti OR 'representable':ab,ti OR 'representation':ab,ti OR 'representation s':ab,ti OR 'representational':ab,ti OR 'representations':ab,ti) AND ('knowledge':ab,ti OR 'knowledge':ab,ti OR 'knowledge s':ab,ti OR 'knowledgeability':ab,ti OR 'knowledgeable':ab,ti OR 'knowledgeably':ab,ti OR 'knowledges':ab,ti)) AND 'Computer':ab,ti) OR 'machine learning':ab,ti OR 'deep learning':ab,ti OR 'supervised machine learning':ab,ti OR 'support vector machine':ab,ti OR 'unsupervised machine learning':ab,ti OR 'diagnosis computer assisted':ab,ti OR 'image interpretation computer assisted':ab,ti)) ) and( ('endoscopes':ab,ti OR ('Colonoscopy':ab,ti OR 'Endoscopy':ab,ti OR 'Gastroscopy':ab,ti OR 'Sigmoidoscopy':ab,ti OR 'Endoscopy':ab,ti OR 'EGDS':ab,ti OR 'esofagogastroduodenoscopy':ab,ti OR 'Colonoscopy':ab,ti OR 'Sigmoidoscopy':ab,ti OR 'proctoscopy':ab,ti OR 'EUS':ab,ti OR 'Endosonography':ab,ti OR ('endoscope':ab,ti AND 'ultrasound':ab,ti))) )and( ( 'meta analysis':ab,ti OR 'Meta-analytic':ab,ti))and( ('Neoplasms':ab,ti OR ('Tumor':ab,ti OR 'Neoplasm':ab,ti OR 'Tumors':ab,ti OR 'Neoplasia':ab,ti OR 'Neoplasias':ab,ti OR 'malignant neoplasm':ab,ti OR 'Malignancy':ab,ti OR 'Malignancies':ab,ti OR 'malignant neoplasms':ab,ti OR 'neoplasm malignant':ab,ti OR 'neoplasms malignant':ab,ti OR 'benign neoplasms':ab,ti OR 'benign neoplasm':ab,ti OR 'neoplasms benign':ab,ti OR 'neoplasm benign':ab,ti) OR 'adenocarcinoma':ab,ti OR ('adenoma malignant':ab,ti OR 'adenomas malignant':ab,ti OR 'malignant adenoma':ab,ti) OR 'pancreas':ab,ti OR ('Pancrea':ab,ti OR 'pancreas':ab,ti) OR ('Stomach':ab,ti OR 'Stomach':ab,ti OR 'gastric':ab,ti) OR ('digestive system':ab,ti OR 'digestive system':ab,ti) OR ('gastrointestinal tract':ab,ti OR 'gastrointestinal tract':ab,ti) OR ('colon':ab,ti OR 'colon':ab,ti OR 'rectum':ab,ti OR 'colorectum':ab,ti OR 'Colorectal':ab,ti) OR ('colorectal neoplasms':ab,ti OR 'colorectal neoplasms':ab,ti) OR ('adenomatous polyposis coli':ab,ti OR 'adenomatous polyposis coli':ab,ti) OR ('Esophagus':ab,ti OR 'Esophagus':ab,ti) OR ('gastrointestinal stromal tumor':ab,ti OR 'gastrointestinal stromal tumors':ab,ti OR 'GIST':ab,ti OR 'GISTS':ab,ti))) |
| Web of science | (("artificial intelligence" OR ("AI" OR "intelligence artificial" OR "computational intelligence"OR "intelligence computational" OR "machine intelligence" OR "intelligence machine"OR "computer reasoning"OR "reasoning computer" OR "computer vision systems" OR "computer vision system"OR "system computer vision" OR "systems computer vision" OR (("vision s" OR "vision, ocular" OR ("Vision" AND "ocular") OR "ocular vision" OR "Vision" OR "visions" OR "visioning") AND "system computer") OR (("education" OR ("knowledge" AND "acquisition") OR "knowledge acquisition") AND "Computer") OR ((("acquisition" OR "acquisitions") AND ("knowledge" OR "knowledge" OR "knowledge s" OR "knowledgeability"OR "knowledgeable" OR "knowledgeably" OR "knowledges")) AND "Computer") OR ((("knowledge" OR "knowledge" OR "knowledge s" OR "knowledgeability" OR "knowledgeable" OR "knowledgeably" OR "knowledges") AND ("representability" OR "representable" OR "representation" OR "representation s" OR "representational"OR "representations")) AND "Computer") OR ((("knowledge" OR "knowledge" OR "knowledge s" OR "knowledgeability" OR "knowledgeable" OR "knowledgeably" OR "knowledges") AND ("representability" OR "representable" OR "representation" OR "representation s" OR "representational" OR "representations")) AND "Computer") OR ((("representability" OR "representable" OR "representation" OR "representation s" OR "representational" OR "representations") AND ("knowledge" OR "knowledge" OR "knowledge s" OR "knowledgeability" OR "knowledgeable" OR "knowledgeably"OR "knowledges")) AND "Computer") OR "machine learning" OR "deep learning" OR "supervised machine learning" OR "support vector machine" OR "unsupervised machine learning" OR "diagnosis computer assisted" OR "image interpretation computer assisted")) ) and( ("endoscopes" OR ("Colonoscopy" OR "Endoscopy" OR "Gastroscopy" OR "Sigmoidoscopy" OR "Endoscopy" OR "EGDS" OR "esofagogastroduodenoscopy" OR "Colonoscopy" OR "Sigmoidoscopy" OR "proctoscopy"OR "EUS"OR "Endosonography" OR ("endoscope"AND "ultrasound"))) )and( ( "meta analysis"OR "Meta-analytic"))and( ("Neoplasms" OR ("Tumor"OR "Neoplasm" OR "Tumors" OR "Neoplasia" OR "Neoplasias" OR "malignant neoplasm" OR "Malignancy" OR "Malignancies" OR "malignant neoplasms" OR "neoplasm malignant" OR "neoplasms malignant" OR "benign neoplasms" OR "benign neoplasm" OR "neoplasms benign"OR "neoplasm benign") OR "adenocarcinoma" OR ("adenoma malignant" OR "adenomas malignant" OR "malignant adenoma") OR "pancreas" OR ("Pancrea" OR "pancreas") OR ("Stomach" OR "Stomach" OR "gastric") OR ("digestive system"OR "digestive system") OR ("gastrointestinal tract" OR "gastrointestinal tract") OR ("colon" OR "colon" OR "rectum" OR "colorectum" OR "Colorectal") OR ("colorectal neoplasms" OR "colorectal neoplasms") OR ("adenomatous polyposis coli" OR "adenomatous polyposis coli") OR ("Esophagus" OR "Esophagus") OR ("gastrointestinal stromal tumor" OR "gastrointestinal stromal tumors" OR "GIST"OR "GISTS"))) |
| Cochrane | ((artificial intelligence:ab,ti OR (AI:ab,ti OR intelligence artificial:ab,ti OR computational intelligence:ab,ti OR intelligence computational:ab,ti OR machine intelligence:ab,ti OR intelligence machine:ab,ti OR computer reasoning:ab,ti OR reasoning computer:ab,ti OR computer vision systems:ab,ti OR computer vision system:ab,ti OR system computer vision:ab,ti OR systems computer vision:ab,ti OR ((vision s:ab,ti OR vision, ocular:ab,ti OR (Vision:ab,ti AND ocular:ab,ti) OR ocular vision:ab,ti OR Vision:ab,ti OR visions:ab,ti OR visioning:ab,ti) AND system computer:ab,ti) OR ((education:ab,ti OR (knowledge:ab,ti AND acquisition:ab,ti) OR knowledge acquisition:ab,ti) AND Computer:ab,ti) OR (((acquisition:ab,ti OR acquisitions:ab,ti) AND (knowledge:ab,ti OR knowledge:ab,ti OR knowledges:ab,ti OR knowledgeability:ab,ti OR knowledgeable:ab,ti OR knowledgeably:ab,ti OR knowledges:ab,ti)) AND Computer:ab,ti) OR (((knowledge:ab,ti OR knowledge:ab,ti OR knowledge s:ab,ti OR knowledgeability:ab,ti OR knowledgeable:ab,ti OR knowledgeably:ab,ti OR knowledges:ab,ti) AND (representability:ab,ti OR representable:ab,ti OR representation:ab,ti OR representation s:ab,ti OR representational:ab,ti OR representations:ab,ti)) AND Computer:ab,ti) OR (((knowledge:ab,ti OR knowledge:ab,ti OR knowledge s:ab,ti OR knowledgeability:ab,ti OR knowledgeable:ab,ti OR knowledgeably:ab,ti OR knowledges:ab,ti) AND (representability:ab,ti OR representable:ab,ti OR representation:ab,ti OR representation s:ab,ti OR representational:ab,ti OR representations:ab,ti)) AND Computer:ab,ti) OR (((representability:ab,ti OR representable:ab,ti OR representation:ab,ti OR representation s:ab,ti OR representational:ab,ti OR representations:ab,ti) AND (knowledge:ab,ti OR knowledge:ab,ti OR knowledge s:ab,ti OR knowledgeability:ab,ti OR knowledgeable:ab,ti OR knowledgeably:ab,ti OR knowledges:ab,ti)) AND Computer:ab,ti) OR machine learning:ab,ti OR deep learning:ab,ti OR supervised machine learning:ab,ti OR support vector machine:ab,ti OR unsupervised machine learning:ab,ti OR diagnosis computer assisted:ab,ti OR image interpretation computer assisted:ab,ti)) ) and( (endoscopes:ab,ti OR (Colonoscopy:ab,ti OR Endoscopy:ab,ti OR Gastroscopy:ab,ti OR Sigmoidoscopy:ab,ti OR Endoscopy:ab,ti OR EGDS:ab,ti OR esofagogastroduodenoscopy:ab,ti OR Colonoscopy:ab,ti OR Sigmoidoscopy:ab,ti OR proctoscopy:ab,ti OR EUS:ab,ti OR Endosonography:ab,ti OR (endoscope:ab,ti AND ultrasound:ab,ti))) )and( ( meta analysis:ab,ti OR Meta-analytic:ab,ti))and( (Neoplasms:ab,ti OR (Tumor:ab,ti OR Neoplasm:ab,ti OR Tumors:ab,ti OR Neoplasia:ab,ti OR Neoplasias:ab,ti OR malignant neoplasm:ab,ti OR Malignancy:ab,ti OR Malignancies:ab,ti OR malignant neoplasms:ab,ti OR neoplasm malignant:ab,ti OR neoplasms malignant:ab,ti OR benign neoplasms:ab,ti OR benign neoplasm:ab,ti OR neoplasms benign:ab,ti OR neoplasm benign:ab,ti) OR adenocarcinoma:ab,ti OR (adenoma malignant:ab,ti OR adenomas malignant:ab,ti OR malignant adenoma:ab,ti) OR pancreas:ab,ti OR (Pancrea:ab,ti OR pancreas:ab,ti) OR (Stomach:ab,ti OR Stomach:ab,ti OR gastric:ab,ti) OR (digestive system:ab,ti OR digestive system:ab,ti) OR (gastrointestinal tract:ab,ti OR gastrointestinal tract:ab,ti) OR (colon:ab,ti OR colon:ab,ti OR rectum:ab,ti OR colorectum:ab,ti OR Colorectal:ab,ti) OR (colorectal neoplasms:ab,ti OR colorectal neoplasms:ab,ti) OR (adenomatous polyposis coli:ab,ti OR adenomatous polyposis coli:ab,ti) OR (Esophagus:ab,ti OR Esophagus:ab,ti) OR (gastrointestinal stromal tumor:ab,ti OR gastrointestinal stromal tumors:ab,ti OR GIST:ab,ti OR GISTS:ab,ti))) |

**Table S 4** Search strategy for individual diagnostic studies in PubMed, EMBASE,Web of Science and Cochrane

| Database | Search strategy |
| --- | --- |
| PUBMED | ("artificial intelligence"[MeSH Terms] OR ("AI"[Title/Abstract] OR "intelligence artificial"[Title/Abstract] OR "computational intelligence"[Title/Abstract] OR "intelligence computational"[Title/Abstract] OR "machine intelligence"[Title/Abstract] OR "intelligence machine"[Title/Abstract] OR "computer reasoning"[Title/Abstract] OR "reasoning computer"[Title/Abstract] OR "computer vision systems"[Title/Abstract] OR "computer vision system"[Title/Abstract] OR "system computer vision"[Title/Abstract] OR "systems computer vision"[Title/Abstract] OR (("vision s"[All Fields] OR "vision, ocular"[MeSH Terms] OR ("Vision"[All Fields] AND "ocular"[All Fields]) OR "ocular vision"[All Fields] OR "Vision"[All Fields] OR "visions"[All Fields] OR "visioning"[All Fields]) AND "system computer"[Title/Abstract]) OR (("education"[MeSH Terms] OR "education"[All Fields] OR ("knowledge"[All Fields] AND "acquisition"[All Fields]) OR "knowledge acquisition"[All Fields]) AND "Computer"[Title/Abstract]) OR ((("acquisition"[All Fields] OR "acquisitions"[All Fields]) AND ("knowledge"[MeSH Terms] OR "knowledge"[All Fields] OR "knowledge s"[All Fields] OR "knowledgeability"[All Fields] OR "knowledgeable"[All Fields] OR "knowledgeably"[All Fields] OR "knowledges"[All Fields])) AND "Computer"[Title/Abstract]) OR ((("knowledge"[MeSH Terms] OR "knowledge"[All Fields] OR "knowledge s"[All Fields] OR "knowledgeability"[All Fields] OR "knowledgeable"[All Fields] OR "knowledgeably"[All Fields] OR "knowledges"[All Fields]) AND ("representability"[All Fields] OR "representable"[All Fields] OR "representation"[All Fields] OR "representation s"[All Fields] OR "representational"[All Fields] OR "representations"[All Fields])) AND "Computer"[Title/Abstract]) OR ((("knowledge"[MeSH Terms] OR "knowledge"[All Fields] OR "knowledge s"[All Fields] OR "knowledgeability"[All Fields] OR "knowledgeable"[All Fields] OR "knowledgeably"[All Fields] OR "knowledges"[All Fields]) AND ("representability"[All Fields] OR "representable"[All Fields] OR "representation"[All Fields] OR "representation s"[All Fields] OR "representational"[All Fields] OR "representations"[All Fields])) AND "Computer"[Title/Abstract]) OR ((("representability"[All Fields] OR "representable"[All Fields] OR "representation"[All Fields] OR "representation s"[All Fields] OR "representational"[All Fields] OR "representations"[All Fields]) AND ("knowledge"[MeSH Terms] OR "knowledge"[All Fields] OR "knowledge s"[All Fields] OR "knowledgeability"[All Fields] OR "knowledgeable"[All Fields] OR "knowledgeably"[All Fields] OR "knowledges"[All Fields])) AND "Computer"[Title/Abstract]) OR "machine learning"[Title/Abstract] OR "deep learning"[Title/Abstract] OR "supervised machine learning"[Title/Abstract] OR "support vector machine"[Title/Abstract] OR "unsupervised machine learning"[Title/Abstract] OR "diagnosis computer assisted"[Title/Abstract] OR "image interpretation computer assisted"[Title/Abstract])) AND ("endoscopes"[MeSH Terms] OR ("Colonoscopy"[Title/Abstract] OR "Endoscopy"[Title/Abstract] OR "Gastroscopy"[Title/Abstract] OR "Sigmoidoscopy"[Title/Abstract] OR "Endoscopy"[Title/Abstract] OR "EGDS"[Title/Abstract] OR "esofagogastroduodenoscopy"[Title/Abstract] OR "Colonoscopy"[Title/Abstract] OR "Sigmoidoscopy"[Title/Abstract] OR "proctoscopy"[Title/Abstract] OR "EUS"[Title/Abstract] OR "Endosonography"[Title/Abstract] OR ("endoscope"[Title/Abstract] AND "ultrasound"[Title/Abstract])))AND ("Neoplasms"[MeSH Terms] OR ("Tumor"[Title/Abstract] OR "Neoplasm"[Title/Abstract] OR "Tumors"[Title/Abstract] OR "Neoplasia"[Title/Abstract] OR "Neoplasias"[Title/Abstract] OR "Cancer"[Title/Abstract] OR "Cancers"[Title/Abstract] OR "malignant neoplasm"[Title/Abstract] OR "Malignancy"[Title/Abstract] OR "Malignancies"[Title/Abstract] OR "malignant neoplasms"[Title/Abstract] OR "neoplasm malignant"[Title/Abstract] OR "neoplasms malignant"[Title/Abstract] OR "benign neoplasms"[Title/Abstract] OR "benign neoplasm"[Title/Abstract] OR "neoplasms benign"[Title/Abstract] OR "neoplasm benign"[Title/Abstract]) OR "adenocarcinoma"[MeSH Terms] OR ("adenoma malignant"[Title/Abstract] OR "adenomas malignant"[Title/Abstract] OR "malignant adenoma"[Title/Abstract]) OR "pancreas"[MeSH Terms] OR ("Pancrea"[Title/Abstract] OR "pancreas"[Title/Abstract]) OR ("Stomach"[MeSH Terms] OR "Stomach"[Title/Abstract] OR "gastric"[Title/Abstract]) OR ("digestive system"[MeSH Terms] OR "digestive system"[Title/Abstract]) OR ("gastrointestinal tract"[MeSH Terms] OR "gastrointestinal tract"[Title/Abstract]) OR ("colon"[MeSH Terms] OR "colon"[Title/Abstract] OR "rectum"[Title/Abstract] OR "colorectum"[Title/Abstract] OR "Colorectal"[Title/Abstract]) OR ("colorectal neoplasms"[MeSH Terms] OR "colorectal neoplasms"[Title/Abstract]) OR ("adenomatous polyposis coli"[MeSH Terms] OR "adenomatous polyposis coli"[Title/Abstract]) OR ("Esophagus"[MeSH Terms] OR "Esophagus"[Title/Abstract]) OR ("gastrointestinal stromal tumor"[Title/Abstract] OR "gastrointestinal stromal tumors"[Title/Abstract] OR "GIST"[Title/Abstract] OR "GISTS"[Title/Abstract])) |
| EMBASE | (('artificial intelligence':ab,ti OR ('AI':ab,ti OR 'intelligence artificial':ab,ti OR 'computational intelligence':ab,ti OR 'intelligence computational':ab,ti OR 'machine intelligence':ab,ti OR 'intelligence machine':ab,ti OR 'computer reasoning':ab,ti OR 'reasoning computer':ab,ti OR 'computer vision systems':ab,ti OR 'computer vision system':ab,ti OR 'system computer vision':ab,ti OR 'systems computer vision':ab,ti OR (('vision s':ab,ti OR 'vision, ocular':ab,ti OR ('Vision':ab,ti AND 'ocular':ab,ti) OR 'ocular vision':ab,ti OR 'Vision':ab,ti OR 'visions':ab,ti OR 'visioning':ab,ti) AND 'system computer':ab,ti) OR (('education':ab,ti OR ('knowledge':ab,ti AND 'acquisition':ab,ti) OR 'knowledge acquisition':ab,ti) AND 'Computer':ab,ti) OR ((('acquisition':ab,ti OR 'acquisitions':ab,ti) AND ('knowledge':ab,ti OR 'knowledge':ab,ti OR 'knowledges':ab,ti OR 'knowledgeability':ab,ti OR 'knowledgeable':ab,ti OR 'knowledgeably':ab,ti OR 'knowledges':ab,ti)) AND 'Computer':ab,ti) OR ((('knowledge':ab,ti OR 'knowledge':ab,ti OR 'knowledge s':ab,ti OR 'knowledgeability':ab,ti OR 'knowledgeable':ab,ti OR 'knowledgeably':ab,ti OR 'knowledges':ab,ti) AND ('representability':ab,ti OR 'representable':ab,ti OR 'representation':ab,ti OR 'representation s':ab,ti OR 'representational':ab,ti OR 'representations':ab,ti)) AND 'Computer':ab,ti) OR ((('knowledge':ab,ti OR 'knowledge':ab,ti OR 'knowledge s':ab,ti OR 'knowledgeability':ab,ti OR 'knowledgeable':ab,ti OR 'knowledgeably':ab,ti OR 'knowledges':ab,ti) AND ('representability':ab,ti OR 'representable':ab,ti OR 'representation':ab,ti OR 'representation s':ab,ti OR 'representational':ab,ti OR 'representations':ab,ti)) AND 'Computer':ab,ti) OR ((('representability':ab,ti OR 'representable':ab,ti OR 'representation':ab,ti OR 'representation s':ab,ti OR 'representational':ab,ti OR 'representations':ab,ti) AND ('knowledge':ab,ti OR 'knowledge':ab,ti OR 'knowledge s':ab,ti OR 'knowledgeability':ab,ti OR 'knowledgeable':ab,ti OR 'knowledgeably':ab,ti OR 'knowledges':ab,ti)) AND 'Computer':ab,ti) OR 'machine learning':ab,ti OR 'deep learning':ab,ti OR 'supervised machine learning':ab,ti OR 'support vector machine':ab,ti OR 'unsupervised machine learning':ab,ti OR 'diagnosis computer assisted':ab,ti OR 'image interpretation computer assisted':ab,ti)) ) and( ('endoscopes':ab,ti OR ('Colonoscopy':ab,ti OR 'Endoscopy':ab,ti OR 'Gastroscopy':ab,ti OR 'Sigmoidoscopy':ab,ti OR 'Endoscopy':ab,ti OR 'EGDS':ab,ti OR 'esofagogastroduodenoscopy':ab,ti OR 'Colonoscopy':ab,ti OR 'Sigmoidoscopy':ab,ti OR 'proctoscopy':ab,ti OR 'EUS':ab,ti OR 'Endosonography':ab,ti OR ('endoscope':ab,ti AND 'ultrasound':ab,ti))) )and( ('Neoplasms':ab,ti OR ('Tumor':ab,ti OR 'Neoplasm':ab,ti OR 'Tumors':ab,ti OR 'Neoplasia':ab,ti OR 'Neoplasias':ab,ti OR 'malignant neoplasm':ab,ti OR 'Malignancy':ab,ti OR 'Malignancies':ab,ti OR 'malignant neoplasms':ab,ti OR 'neoplasm malignant':ab,ti OR 'neoplasms malignant':ab,ti OR 'benign neoplasms':ab,ti OR 'benign neoplasm':ab,ti OR 'neoplasms benign':ab,ti OR 'neoplasm benign':ab,ti) OR 'adenocarcinoma':ab,ti OR ('adenoma malignant':ab,ti OR 'adenomas malignant':ab,ti OR 'malignant adenoma':ab,ti) OR 'pancreas':ab,ti OR ('Pancrea':ab,ti OR 'pancreas':ab,ti) OR ('Stomach':ab,ti OR 'Stomach':ab,ti OR 'gastric':ab,ti) OR ('digestive system':ab,ti OR 'digestive system':ab,ti) OR ('gastrointestinal tract':ab,ti OR 'gastrointestinal tract':ab,ti) OR ('colon':ab,ti OR 'colon':ab,ti OR 'rectum':ab,ti OR 'colorectum':ab,ti OR 'Colorectal':ab,ti) OR ('colorectal neoplasms':ab,ti OR 'colorectal neoplasms':ab,ti) OR ('adenomatous polyposis coli':ab,ti OR 'adenomatous polyposis coli':ab,ti) OR ('Esophagus':ab,ti OR 'Esophagus':ab,ti) OR ('gastrointestinal stromal tumor':ab,ti OR 'gastrointestinal stromal tumors':ab,ti OR 'GIST':ab,ti OR 'GISTS':ab,ti))) |
| Web of science | (("artificial intelligence" OR ("AI" OR "intelligence artificial" OR "computational intelligence"OR "intelligence computational" OR "machine intelligence" OR "intelligence machine"OR "computer reasoning"OR "reasoning computer" OR "computer vision systems" OR "computer vision system"OR "system computer vision" OR "systems computer vision" OR (("vision s" OR "vision, ocular" OR ("Vision" AND "ocular") OR "ocular vision" OR "Vision" OR "visions" OR "visioning") AND "system computer") OR (("education" OR ("knowledge" AND "acquisition") OR "knowledge acquisition") AND "Computer") OR ((("acquisition" OR "acquisitions") AND ("knowledge" OR "knowledge" OR "knowledge s" OR "knowledgeability"OR "knowledgeable" OR "knowledgeably" OR "knowledges")) AND "Computer") OR ((("knowledge" OR "knowledge" OR "knowledge s" OR "knowledgeability" OR "knowledgeable" OR "knowledgeably" OR "knowledges") AND ("representability" OR "representable" OR "representation" OR "representation s" OR "representational"OR "representations")) AND "Computer") OR ((("knowledge" OR "knowledge" OR "knowledge s" OR "knowledgeability" OR "knowledgeable" OR "knowledgeably" OR "knowledges") AND ("representability" OR "representable" OR "representation" OR "representation s" OR "representational" OR "representations")) AND "Computer") OR ((("representability" OR "representable" OR "representation" OR "representation s" OR "representational" OR "representations") AND ("knowledge" OR "knowledge" OR "knowledge s" OR "knowledgeability" OR "knowledgeable" OR "knowledgeably"OR "knowledges")) AND "Computer") OR "machine learning" OR "deep learning" OR "supervised machine learning" OR "support vector machine" OR "unsupervised machine learning" OR "diagnosis computer assisted" OR "image interpretation computer assisted")) ) and( ("endoscopes" OR ("Colonoscopy" OR "Endoscopy" OR "Gastroscopy" OR "Sigmoidoscopy" OR "Endoscopy" OR "EGDS" OR "esofagogastroduodenoscopy" OR "Colonoscopy" OR "Sigmoidoscopy" OR "proctoscopy"OR "EUS"OR "Endosonography" OR ("endoscope"AND "ultrasound"))) )and( ("Neoplasms" OR ("Tumor"OR "Neoplasm" OR "Tumors" OR "Neoplasia" OR "Neoplasias" OR "malignant neoplasm" OR "Malignancy" OR "Malignancies" OR "malignant neoplasms" OR "neoplasm malignant" OR "neoplasms malignant" OR "benign neoplasms" OR "benign neoplasm" OR "neoplasms benign"OR "neoplasm benign") OR "adenocarcinoma" OR ("adenoma malignant" OR "adenomas malignant" OR "malignant adenoma") OR "pancreas" OR ("Pancrea" OR "pancreas") OR ("Stomach" OR "Stomach" OR "gastric") OR ("digestive system"OR "digestive system") OR ("gastrointestinal tract" OR "gastrointestinal tract") OR ("colon" OR "colon" OR "rectum" OR "colorectum" OR "Colorectal") OR ("colorectal neoplasms" OR "colorectal neoplasms") OR ("adenomatous polyposis coli" OR "adenomatous polyposis coli") OR ("Esophagus" OR "Esophagus") OR ("gastrointestinal stromal tumor" OR "gastrointestinal stromal tumors" OR "GIST"OR "GISTS"))) |
| Cochrane | ((artificial intelligence:ab,ti OR (AI:ab,ti OR intelligence artificial:ab,ti OR computational intelligence:ab,ti OR intelligence computational:ab,ti OR machine intelligence:ab,ti OR intelligence machine:ab,ti OR computer reasoning:ab,ti OR reasoning computer:ab,ti OR computer vision systems:ab,ti OR computer vision system:ab,ti OR system computer vision:ab,ti OR systems computer vision:ab,ti OR ((vision s:ab,ti OR vision, ocular:ab,ti OR (Vision:ab,ti AND ocular:ab,ti) OR ocular vision:ab,ti OR Vision:ab,ti OR visions:ab,ti OR visioning:ab,ti) AND system computer:ab,ti) OR ((education:ab,ti OR (knowledge:ab,ti AND acquisition:ab,ti) OR knowledge acquisition:ab,ti) AND Computer:ab,ti) OR (((acquisition:ab,ti OR acquisitions:ab,ti) AND (knowledge:ab,ti OR knowledge:ab,ti OR knowledges:ab,ti OR knowledgeability:ab,ti OR knowledgeable:ab,ti OR knowledgeably:ab,ti OR knowledges:ab,ti)) AND Computer:ab,ti) OR (((knowledge:ab,ti OR knowledge:ab,ti OR knowledge s:ab,ti OR knowledgeability:ab,ti OR knowledgeable:ab,ti OR knowledgeably:ab,ti OR knowledges:ab,ti) AND (representability:ab,ti OR representable:ab,ti OR representation:ab,ti OR representation s:ab,ti OR representational:ab,ti OR representations:ab,ti)) AND Computer:ab,ti) OR (((knowledge:ab,ti OR knowledge:ab,ti OR knowledge s:ab,ti OR knowledgeability:ab,ti OR knowledgeable:ab,ti OR knowledgeably:ab,ti OR knowledges:ab,ti) AND (representability:ab,ti OR representable:ab,ti OR representation:ab,ti OR representation s:ab,ti OR representational:ab,ti OR representations:ab,ti)) AND Computer:ab,ti) OR (((representability:ab,ti OR representable:ab,ti OR representation:ab,ti OR representation s:ab,ti OR representational:ab,ti OR representations:ab,ti) AND (knowledge:ab,ti OR knowledge:ab,ti OR knowledge s:ab,ti OR knowledgeability:ab,ti OR knowledgeable:ab,ti OR knowledgeably:ab,ti OR knowledges:ab,ti)) AND Computer:ab,ti) OR machine learning:ab,ti OR deep learning:ab,ti OR supervised machine learning:ab,ti OR support vector machine:ab,ti OR unsupervised machine learning:ab,ti OR diagnosis computer assisted:ab,ti OR image interpretation computer assisted:ab,ti)) ) and( (endoscopes:ab,ti OR (Colonoscopy:ab,ti OR Endoscopy:ab,ti OR Gastroscopy:ab,ti OR Sigmoidoscopy:ab,ti OR Endoscopy:ab,ti OR EGDS:ab,ti OR esofagogastroduodenoscopy:ab,ti OR Colonoscopy:ab,ti OR Sigmoidoscopy:ab,ti OR proctoscopy:ab,ti OR EUS:ab,ti OR Endosonography:ab,ti OR (endoscope:ab,ti AND ultrasound:ab,ti))) )and( (Neoplasms:ab,ti OR (Tumor:ab,ti OR Neoplasm:ab,ti OR Tumors:ab,ti OR Neoplasia:ab,ti OR Neoplasias:ab,ti OR malignant neoplasm:ab,ti OR Malignancy:ab,ti OR Malignancies:ab,ti OR malignant neoplasms:ab,ti OR neoplasm malignant:ab,ti OR neoplasms malignant:ab,ti OR benign neoplasms:ab,ti OR benign neoplasm:ab,ti OR neoplasms benign:ab,ti OR neoplasm benign:ab,ti) OR adenocarcinoma:ab,ti OR (adenoma malignant:ab,ti OR adenomas malignant:ab,ti OR malignant adenoma:ab,ti) OR pancreas:ab,ti OR (Pancrea:ab,ti OR pancreas:ab,ti) OR (Stomach:ab,ti OR Stomach:ab,ti OR gastric:ab,ti) OR (digestive system:ab,ti OR digestive system:ab,ti) OR (gastrointestinal tract:ab,ti OR gastrointestinal tract:ab,ti) OR (colon:ab,ti OR colon:ab,ti OR rectum:ab,ti OR colorectum:ab,ti OR Colorectal:ab,ti) OR (colorectal neoplasms:ab,ti OR colorectal neoplasms:ab,ti) OR (adenomatous polyposis coli:ab,ti OR adenomatous polyposis coli:ab,ti) OR (Esophagus:ab,ti OR Esophagus:ab,ti) OR (gastrointestinal stromal tumor:ab,ti OR gastrointestinal stromal tumors:ab,ti OR GIST:ab,ti OR GISTS:ab,ti))) |


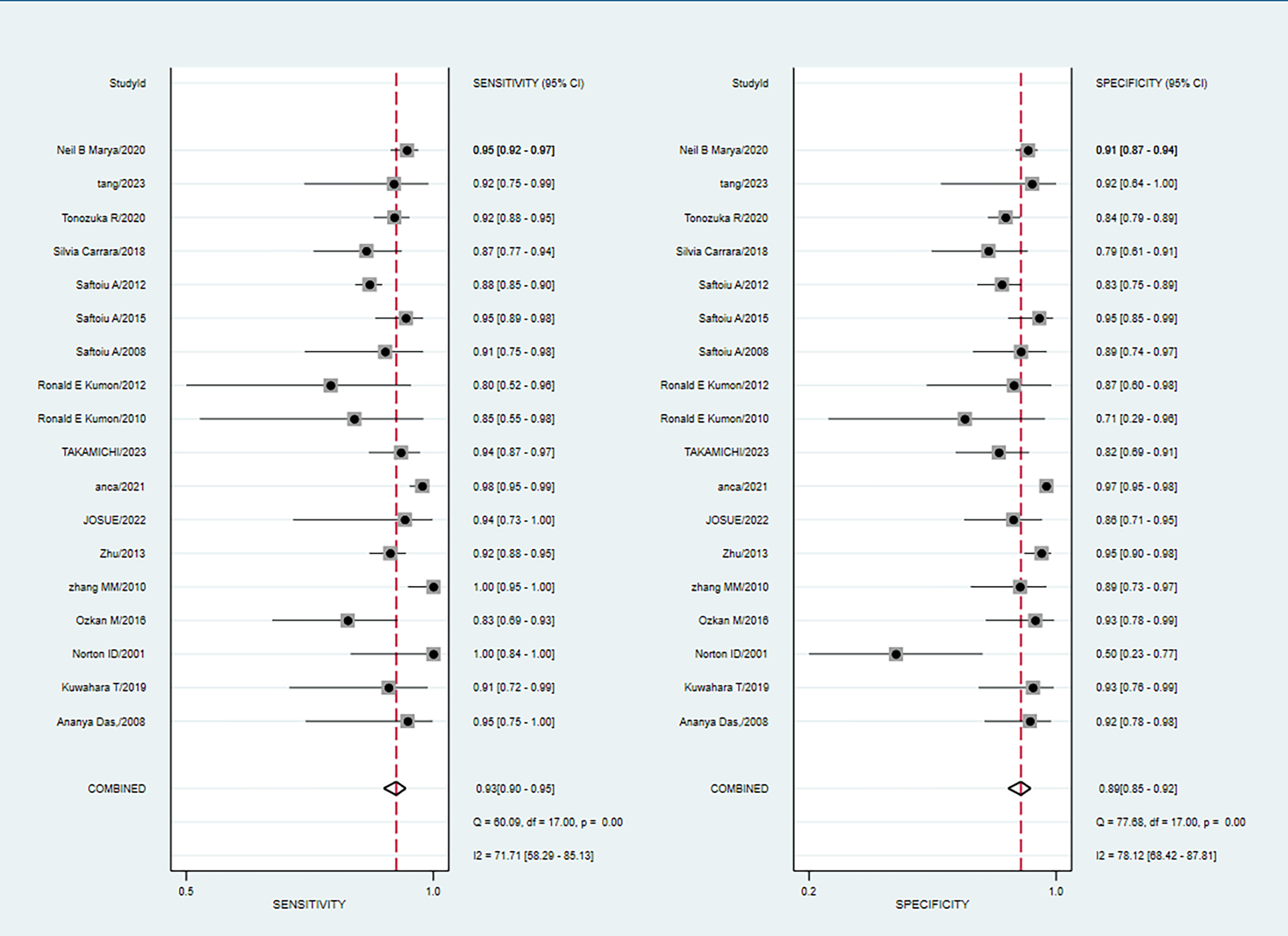


**Figure S 2** Pooled sensitivity and specificity of AI-assisted endoscopy in the diagnosis of Pancreatic Cancer


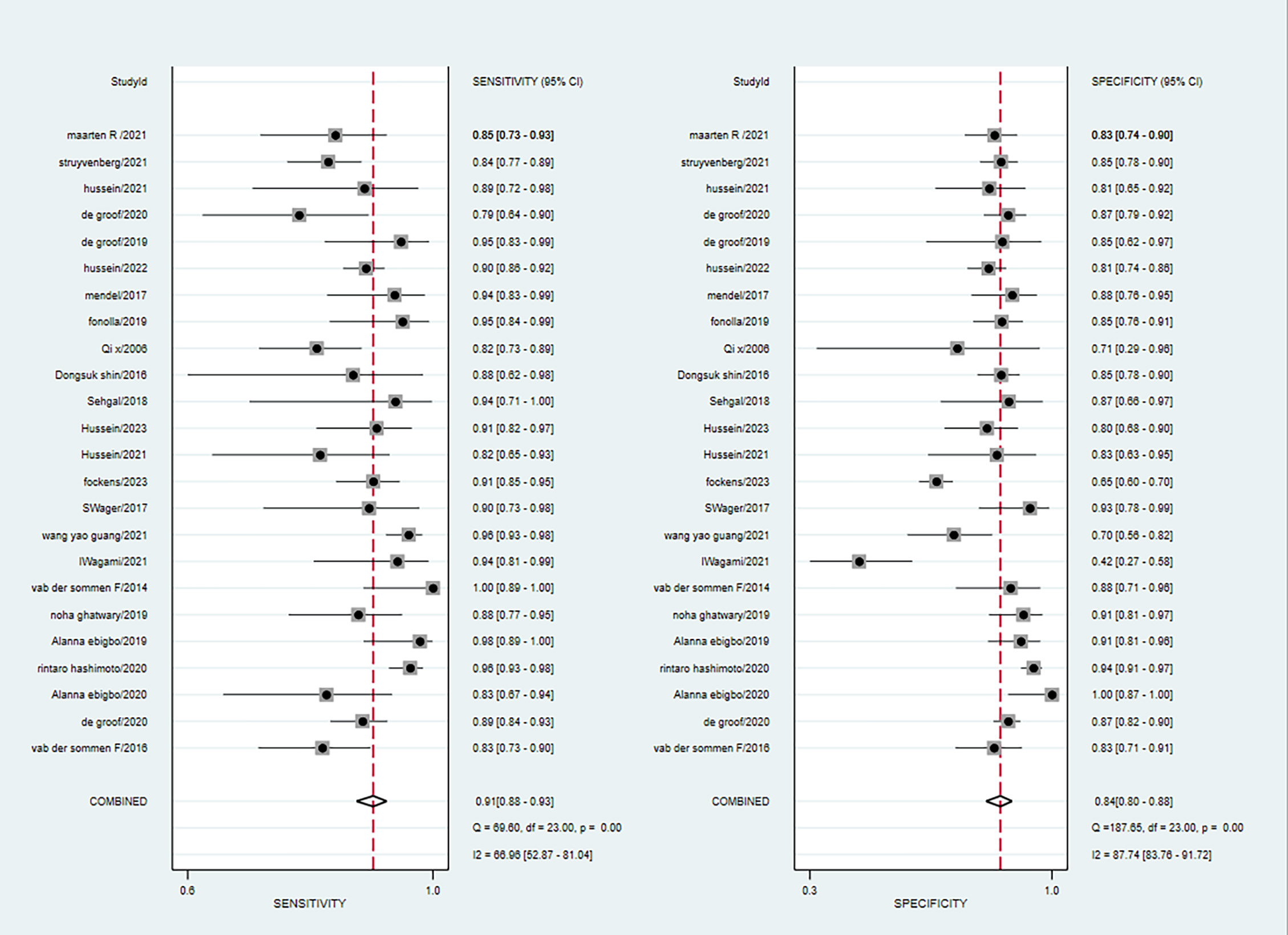


**Figure S 3** Pooled sensitivity and specificity of AI-assisted endoscopy in the diagnosis of Barrett's esophageal adenocarcinoma


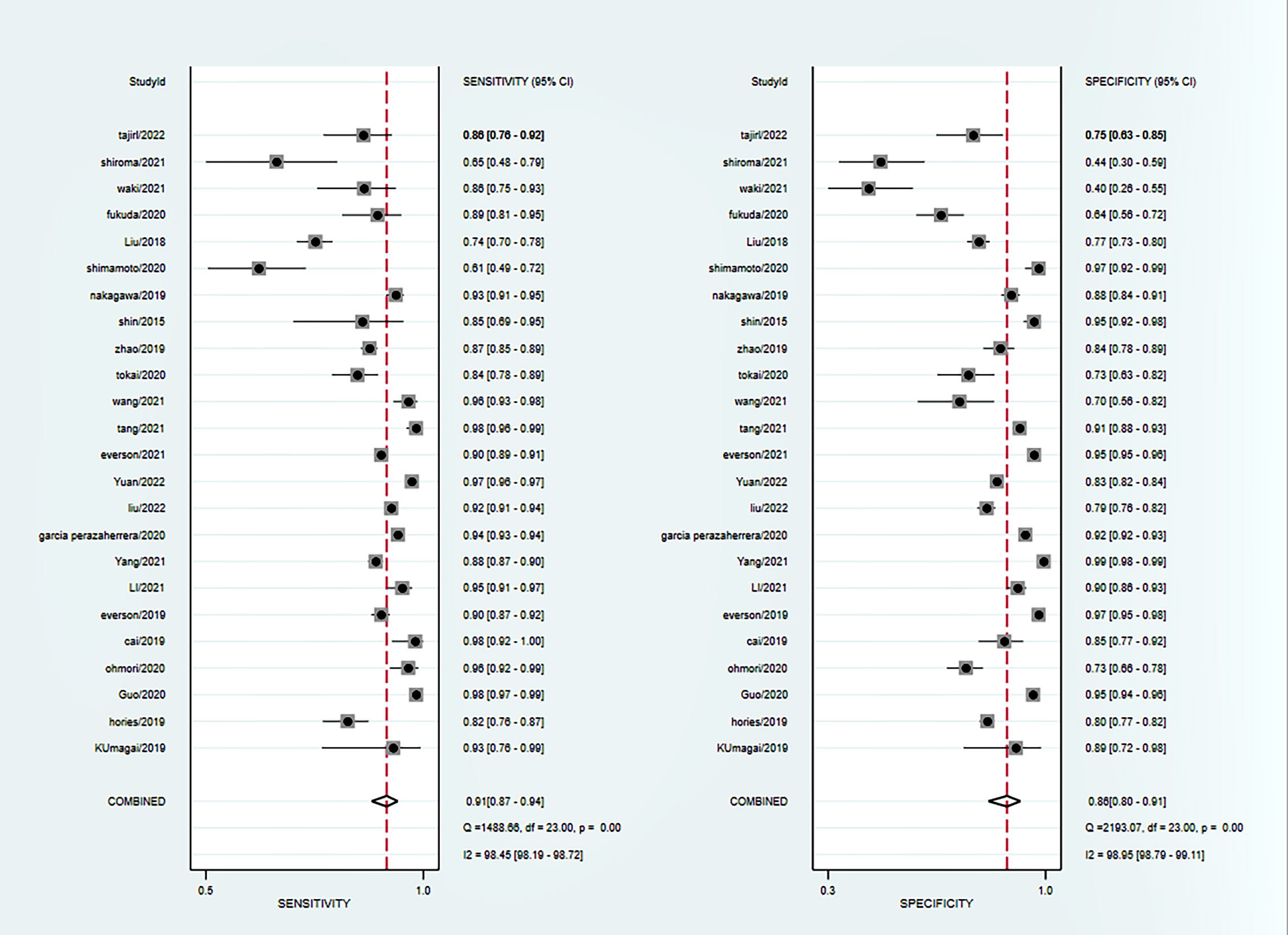


**Figure S 4** Pooled sensitivity and specificity of AI-assisted endoscopy in diagnosing Esophageal squamous cell carcinoma


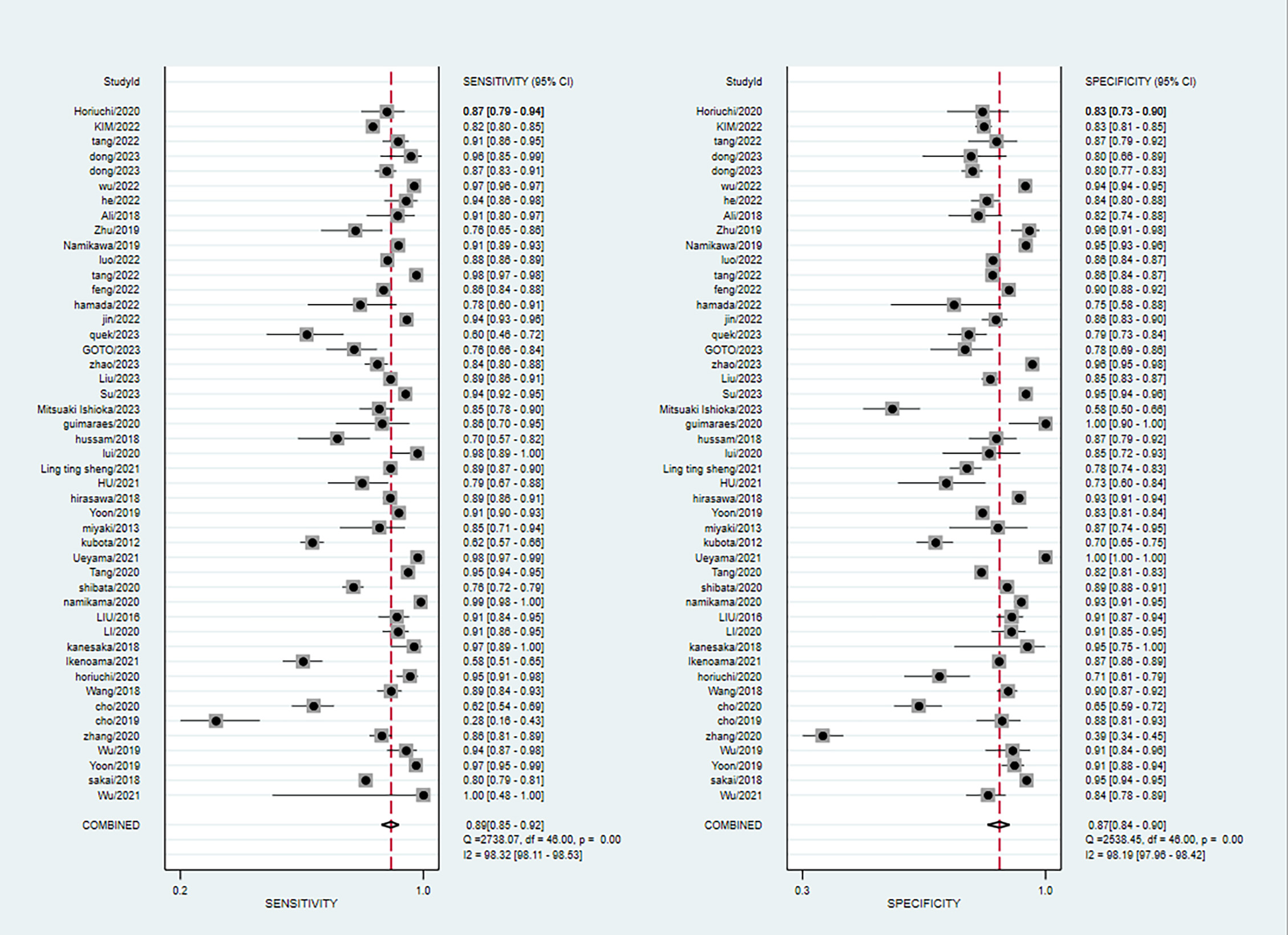


**Figure S 5** Pooled sensitivity and specificity of AI-assisted endoscopy in the diagnosis of Gastric cancer


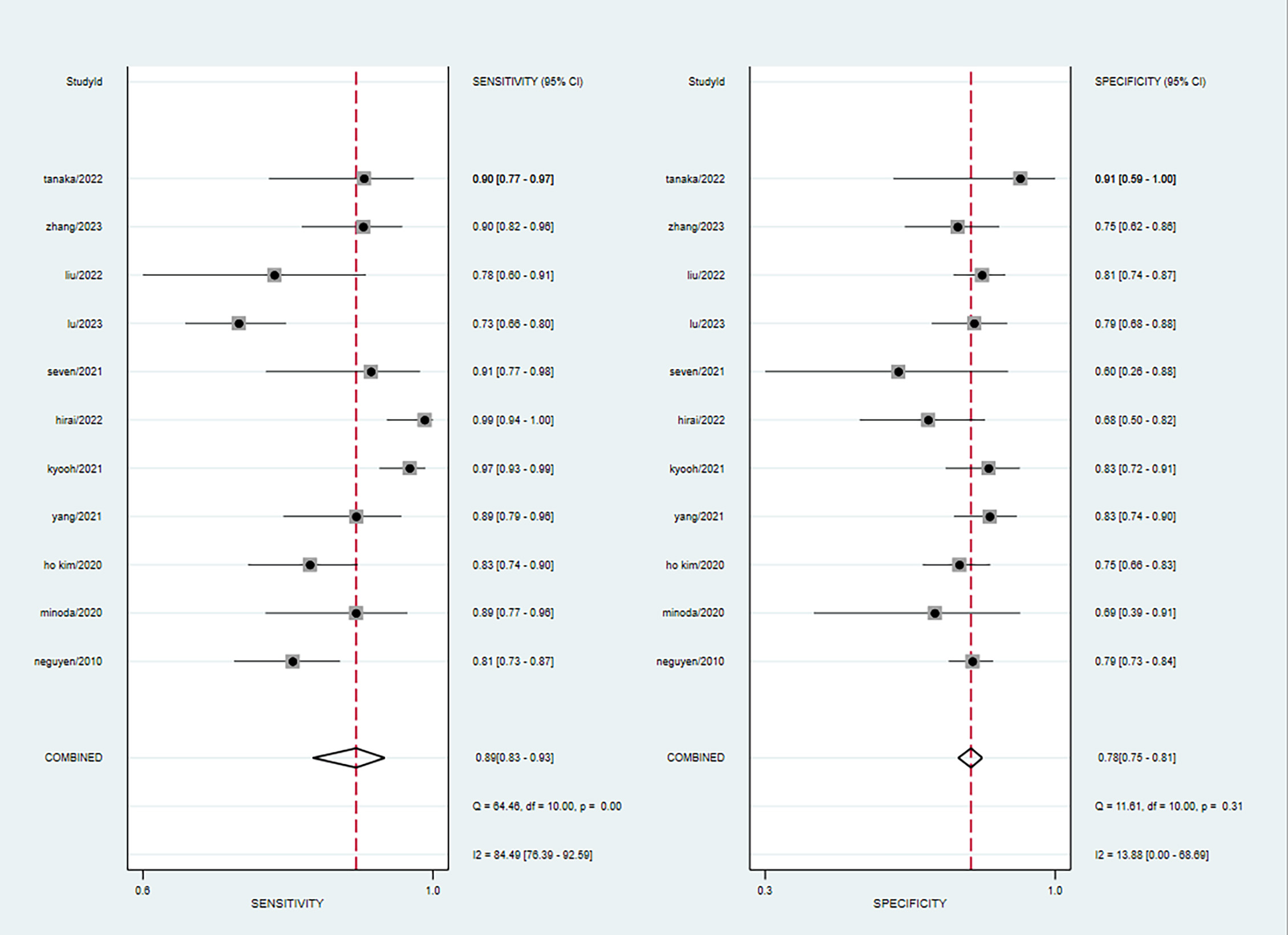


**Figure S 6** Pooled sensitivity and specificity of AI-assisted endoscopy in the diagnosis of mesenchymal stromal tumor


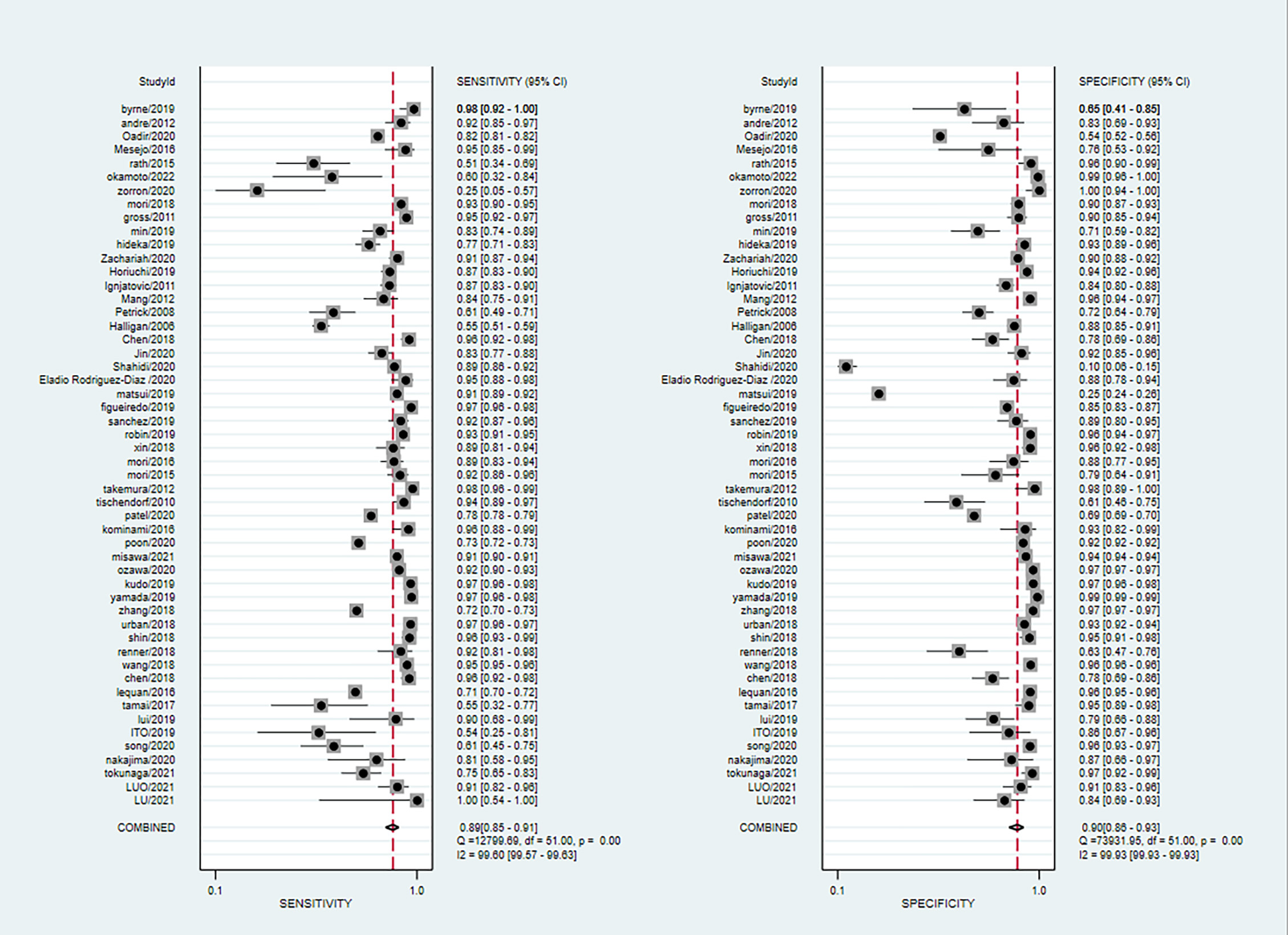


**Figure S 7** Pooled sensitivity and specificity of AI-assisted endoscopy in the diagnosis of Coloretal cancer


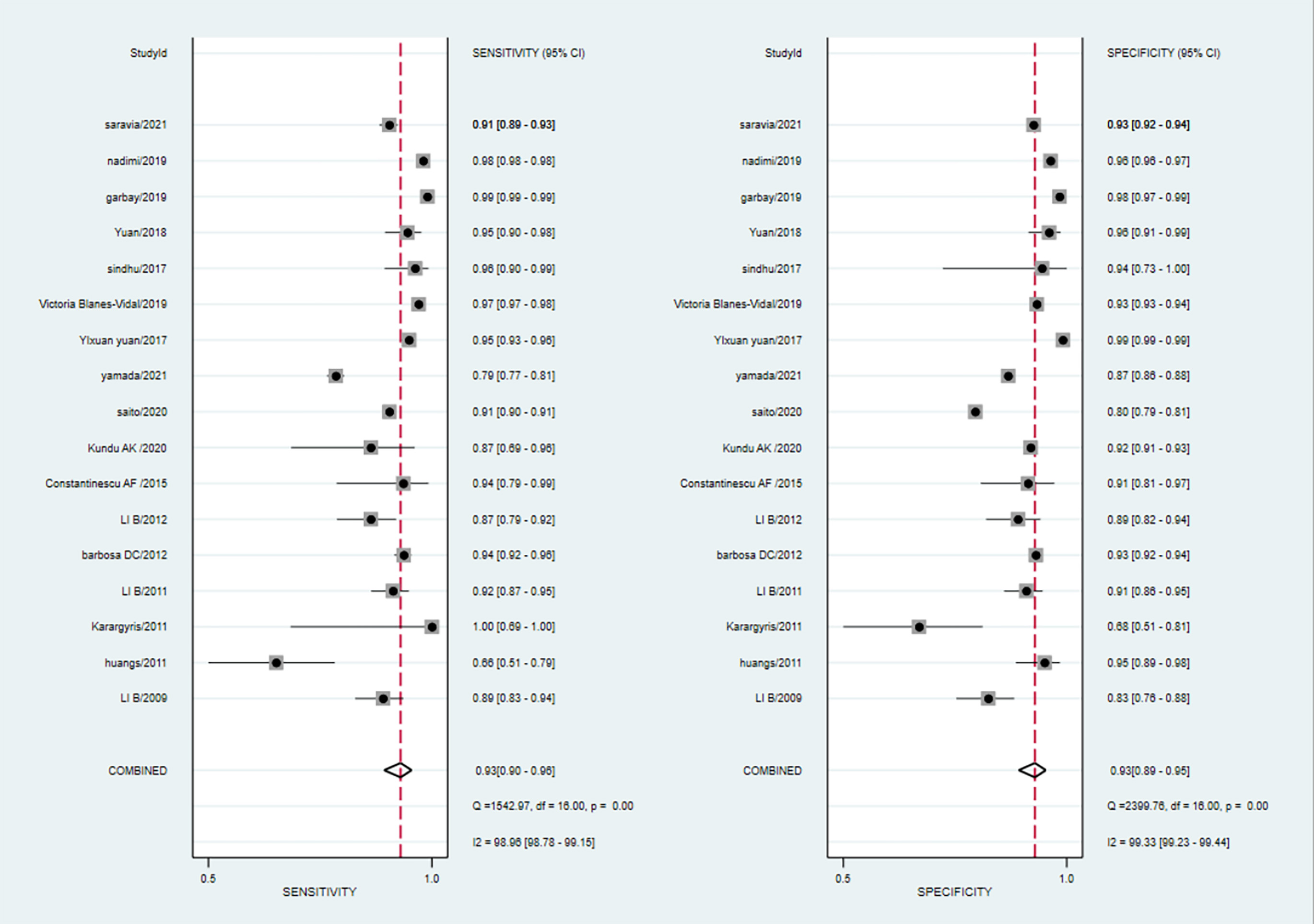


**Figure S 8** Pooled sensitivity and specificity of AI-assisted WCE in the diagnosis of gastrointestinal tumors
